# The Impact of Location and Device Coupling on the Performance of the Osia System Actuator

**DOI:** 10.1155/2022/9079903

**Published:** 2022-04-02

**Authors:** Guy Fierens, Charlotte Borgers, Tristan Putzeys, Joris Walraevens, Astrid Van Wieringen, Nicolas Verhaert

**Affiliations:** ^1^KU Leuven-University of Leuven, Department of Neurosciences, Exporl, B-3000 Leuven, Belgium; ^2^Cochlear Technology Centre, Mechelen, Belgium; ^3^KU Leuven-University of Leuven, Department of Physics and Astronomy, Laboratory for Soft Matter and Biophysics, Heverlee, Belgium; ^4^University Hospitals Leuven, Department of Otorhinolaryngology, Head and Neck Surgery, Leuven, Belgium

## Abstract

Active transcutaneous bone conduction (BC) devices offer the benefit of improved power output compared to passive transcutaneous devices and remove the risk of skin infections that are more common in traditional percutaneous BC devices. Despite these advantages, more research is needed on implant location, device coupling, and their influence on device performance. This study is aimed at quantifying the extent to which certain parameters affect device output when using the Osia® system actuator. Parameters under study are (1) implant location, (2) comparison with the actuator of a state-of-the-art BC device, (3) bone undergrowth simulation, and (4) skull fixation. Five human cadaveric heads were implanted with the actuator at three different implant locations: (1) recommended, (2) posterior Osia® positions, and (3) standard Baha® position. At each location, the cochlear promontory velocity and the intracochlear pressure difference were measured. A percutaneous bone conduction actuator was used as a reference for the obtained measurements. Stimulation levels corresponded to a hearing level of 60 dB HL for frequencies between 250 and 6000 Hz. In addition, bone cement was used as a simulation for reactive bone growth. Results obtained in four heads indicate an improved power transmission of the transcutaneous actuator when implanted at the recommended position compared to the actuator of the percutaneous device on its respective recommended location when stimulating at an identical force level. A correlation was found between the promontory vibration and the actuator position, indicating that the same level of stimulation leads to higher promontory vibrations when the device is implanted closer to the ear canal. This is mainly reflected at frequencies higher than 1 kHz, where an increase was observed in both measurement modalities. At lower frequencies (<1 kHz), the power transmission is less influenced by the implant position and differences between the acquired responses are limited. In addition, when no rigid coupling to the skull is provided, power transfer losses of up to 30 dB can be expected.

## 1. Introduction

Bone conduction (BC) stimulation is used to rehabilitate the hearing function in patients with conductive or mixed hearing loss or for patients with single-sided deafness. Until the release of the first commercial active transcutaneous bone conduction implant (BCI) BoneBridge from Med-El in 2012, two other types of BCI had been available: passive percutaneous and passive transcutaneous BC devices. An investigational transcutaneous BCI that is not yet commercially available is being developed, based on the early work by Hakansson et al. [1, 2]. With a percutaneous system, an abutment is implanted on a bone screw through the skin to connect the BC device to the bony skull. Sound is thus transmitted via a stiff mechanical coupling, which ensures a good connection, powerful enough to rehabilitate hearing losses up to 65 dB HL sensorineural hearing loss [3]. Other advantages of percutaneous systems are good retention of the sound processor due to a good coupling with the abutment and a maximal hearing performance due to the direct coupling to the skull. Limitations of these systems are a potential higher prevalence of skin infections, the need for aftercare and good hygiene, and also the visibility due to the larger sound processor that is needed for more powerful options. For passive transcutaneous BCIs, sound is transmitted through the skin without penetration, which significantly reduces the risk of skin infections. Advantages of this system are improved aesthetics through a smaller sound processor with a lower protrusion, maximal ease of use, minimal need for aftercare, and reduced risk of skin infections. However, passive transcutaneous systems are only suitable for patients with moderate hearing losses as the output of the system drops above 1-3 kHz due to skin attenuation [4, 5]. To overcome the shortcomings of these BCIs and to better meet the customer needs, active transcutaneous solutions like the BoneBridge have been developed. Recently, a novel active osseointegrated steady-state implant (OSI) has been developed by Cochlear Ltd. as part of the Osia® OSI200 system. The OSI200 actuator is implanted behind the pinna on the mastoid portion of the temporal bone. This location is distinctly different compared to state-of-the-art BC devices, such as passive transcutaneous or percutaneous systems which are implanted posterosuperior to the pinna. By using a transcutaneous inductive link, the actuator can be placed closer to the ear canal while the sound processor can be placed at the superior level of the pinna, which is optimal for microphone placement.

As the implantation of the OSI200 actuator is more invasive compared to state-of-the-art passive BC devices, preclinical evaluation of the system is required. In a recent study by Dobrev et al. (2018), the effect of position was examined by measuring the three-dimensional promontory velocity on the ipsilateral and contralateral side in Thiel-embalmed cadaveric heads. Furthermore, the effect of a mastoidectomy on the transfer function efficacy was tested by repeating the measurements on the ipsilateral side after performing a mastoidectomy. Results showed a higher promontory response at a position close to the ear canal compared to the standard Baha location [6]. It has also been confirmed by others that the position closest to the cochlea generates the most efficient sound transmission [7–9]. A transmission loss with increasing distance from the cochlea was also reported by Stenfelt and Goode [10].

A more complex measure to characterize acoustic stimulation is the complex pressure difference between the cochlear scala tympani and vestibuli. The interest in this measure originated several decades ago, with the first study in the guinea pig being reported in 1963 [11]. The technique allows quantifying the driver for auditory transduction in the form of the complex pressure difference [12, 13]. Prior research also showed that the phase and magnitude response of the complex pressure difference is close to identical to the neurophysiological cochlear microphonic recorded near the same cochlear location [14]. Since its first reporting, efforts have mainly been focused on optimizing and utilizing the technique for air conduction stimulation [12, 13] or forward stimulation with middle ear devices [15]. Valuable insights regarding nonlinearity [16], the correlation between scala vestibuli pressure and ear canal pressure [17], the contribution of the two scalar pressures on the pressure difference [15], the exploration of sensor technologies [18–20], and the definition of proper experimental procedures [12, 21] were obtained during this period. Only recently, researchers have started looking into using the technique for bone conduction stimulation as well [22, 23], as measurement results indicate that vibrational artifacts can be limited with proper sensor fixation.

The results of the abovementioned preclinical studies assume a rigid and stiff coupling of the device to the patient's anatomy, as recommended by device manufacturers. A recent study by Dolhen et al. questioned this state-of-the-art by using a minimally invasive pocket surgical technique without rigid fixation in ten Baha® Attract patients [24]. The study reported improvements in audiological results after three months and no issues with device displacement or removal after two years. For the Osia system, the manufacturer recommends flattening the bone under the actuator to avoid interference between the bone and the device. Interference that is not cleared before mounting the device could create a mechanical lever that impedes the optimal alignment, reducing device efficiency.

In this study, the effect of the anatomical location of the device on its performance is investigated together with the importance of device coupling. This is done by measuring the vibrational response of the cochlear promontory at lower and clinically more relevant stimulation levels compared to a prior study using this device [6]. As an additional measure of performance, intracochlear pressure (ICP) measurements will be performed, as they are hypothesized to be an accurate measure for investigating cochlear transmission with BC stimulation [22, 23]. As a secondary goal, the incidence of bone undergrowth under the implanted actuator is considered a simulation model to investigate any degrading effects on efficiency due to device-bone interference. By combining the use of intracochlear pressure and promontory velocity, we aim to provide a specific test framework for quantifying the output of this novel active transcutaneous bone conduction actuator.

The objectives of this study are as follows:

1. To define the most optimal implant location of the OSI200 actuator for different stimulation frequencies
2. To compare the output of the OSI200 actuator with the output of a state-of-the-art percutaneous BC device (Baha 5 SuperPower) based on their respective implant locations when stimulating with an identical force level
3. To simulate and study the effect of cortical bone undergrowth under the OSI200 actuator
4. To compare the efficiency of sound transmission of the OSI200 actuator without a rigid coupling to the skull

## 2. Materials and Methods

### 2.1. Specimen Preparation

Human cadaveric specimens were provided by the Vesalius Institute of the University of Leuven following ethical approval of the same institute (S65502). Five full head specimens were used during the experiment. The specimens were harvested and refrigerated within the first 72 hours postmortem, complying with the guidelines defined in technical standard ASTM F2504-05 [25].

**Figure 1:**
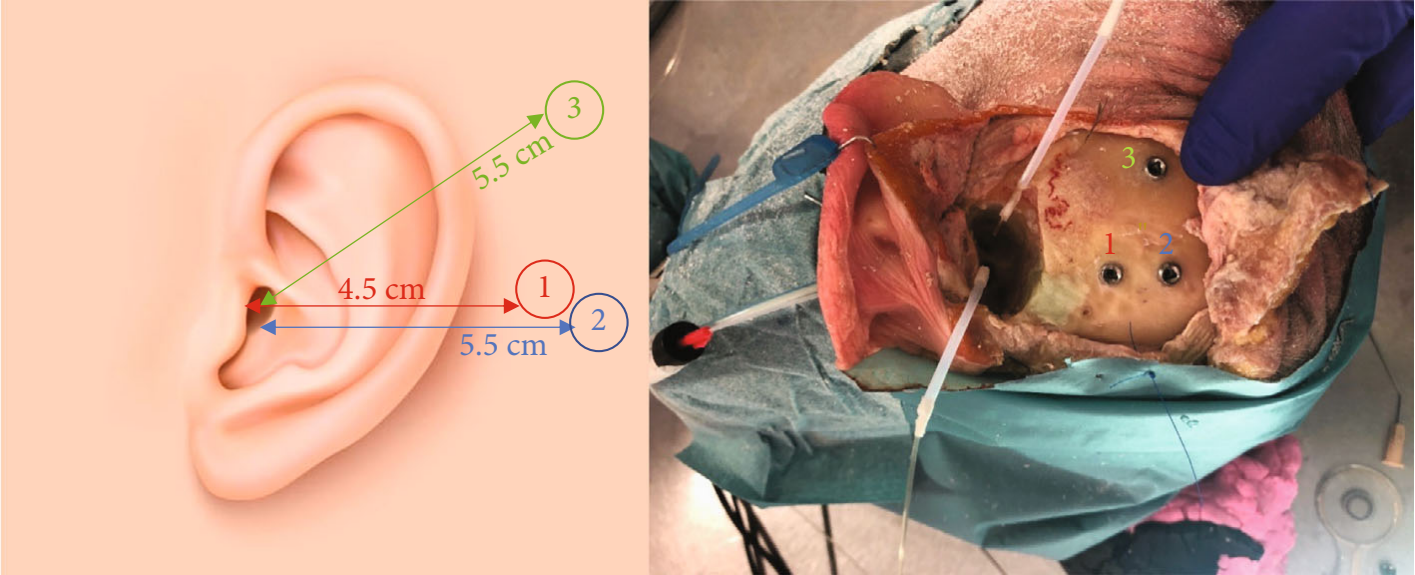
Indication of the three different positions investigated during the experiment: (1) the postauricular Osia position, (2) the postauricular Osia position for single-sided deafness, and (3) the recommended position for Baha devices.

The external ear canal and tympanic membrane were inspected before the start of the experiment to exclude abnormalities. Surgical preparation of the experimental site was performed using a mastoidectomy and an enlarged posterior tympanotomy in order to expose the middle ear. Access to the middle ear was maximized by partly removing the mastoidal portion of the facial nerve. After thinning of the cochlear wall around the scala vestibuli and tympani, the location of the bone screws was annotated on the skull using a permanent marker. Three actuator locations were investigated (Figure 1).

1. The recommended postauricular Osia OSI200 surgical position, at a distance of 4.5 cm with respect to the ear canal due to the necessary mastoidectomy
2. The recommended Osia OSI200 surgical position for single-sided deafness, where the actuator is implanted more posteriorly to allow proper sound conduction to the contralateral ear. During the experiments, this distance was fixed at 5.5 cm posterior to the center of the ear canal
3. The recommended surgical position for percutaneous Baha actuators, located at 5.5 cm posterosuperior to the center of the external ear canal at an angle of approximately 40° with respect to a line connecting both prior positions

### 2.2. Stimulation and Signal Acquisition

#### 2.2.1. Quality Control

**Figure 2:**
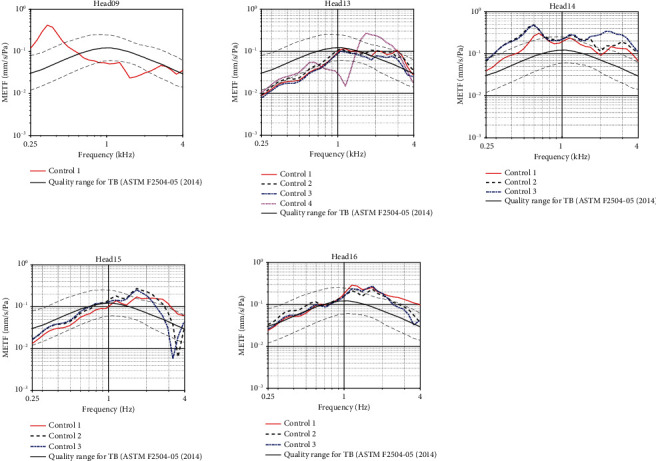
Summary of initial and intermediate control measurements for all specimens. Measured curves are shown in red, black, pink, or blue depending on the measurement time. Reference values obtained from ASTM F2504-05 are illustrated by dotted black lines.

Before the start of each experiment, the quality of the specimen was verified using the procedure described in ASTM F2504-05 [25] for temporal bones. For these measurements, the specimens were suspended on a ring made of modeling clay (Play-Doh, Hasbro, Pawtucket, USA), providing mechanical insulation from a vibration-isolated table (M-VIS3048-SG2-325A, Newport Spectra-Physics, Utrecht, the Netherlands). A ring of modelling clay with an average thickness of 4 cm was placed on top of the table surface with a circumference that would encircle the contralateral ear. The head specimen was then placed on its side with the contralateral side contacting the modelling clay, ensuring that the specimen itself was not touching the table. This technique is similar to the technique used by other authors to fixate a full head specimen [26, 27]. While this is not representative for rigid body-like motion, it does not interfere with transverse travelling waves. A stepped-sine sweep was presented to the specimen's external ear canal using an insert phone (ER3•C, Etymotic Research, Illinois, USA) at a sound level of approximately 102.5 dB SPL at 50 logarithmically spaced frequencies between 100 and 10.000 Hz. Using a sound card (Fireface UC, RME, Haimhausen DE) with custom software RBA [28], the stimulation was applied while simultaneously measuring the pressure in the ear canal and the velocity of the stapes. Ear canal pressure was measured using a probe microphone (ER7, Etymotic Research, Illinois, USA). The velocity of the stapes was measured by aiming the laser beam of a 1D laser Doppler vibrometer system (LDV; OFV-534 Compact Sensor Head and A-HLV MM 30 Micromanipulator; OFV 5000 Vibrometer controller; Polytec GmbH, Waldbronn, Germany) at the posterior crus equipped with a piece of retroreflective tape (A-RET-T010, Polytec GmbH, Waldbronn, Germany). No corrections were made for the angle between the laser beam and the posterior crus as the angle was approximately normal to the motion direction in all specimens. The middle ear transfer function (METF) was calculated by taking the ratio between the stapes velocity and the pressure in the ear canal. To evaluate the functionality of the middle ear, the calculated METF was compared to the range defined by Rosowski et al. [29] for frequencies between 0.25 and 4 kHz [25]. The METF remained between these ranges in four out of five specimens. These were included for further study. Between subsequent control measurements, differences in METF remained between 4 dB for all specimens for most frequencies. This is in line with the findings of other authors [13]. Measured curves for each specimen are shown in Figure 2.

#### 2.2.2. Performance Measurements

Stimulation signals used during the experiment were generated by a sound card connected to an LPA01 amplifier set to a unit gain (Newtons4th Ltd., Leicester, UK) to ensure a constant power output. During intermediate checkpoints, the sound card was also used to measure the ear canal pressure and stapes vibrations according to the procedure described above at a sampling rate of 96 kHz. During other experimental steps, the soundcard was used solely for stimulation and lock-in amplifiers (SR830, Stanford Research Systems, California, USA) were used to improve the signal-to-noise ratio (SNR) at lower stimulation intensities. The lock-in amplifier leverages the knowledge that noise is spread out over a wider range of frequencies than the signal of interest. It does so by using phase-sensitive detection at the frequency of a reference signal that is fed to the amplifier [30–32]. In our case, the stimulation signal was used as a reference. Stimulation signals were 80 seconds long and contained a single frequency sine signal to stimulate the specimen and serve as a reference signal for the lock-in amplifier. The lock-in amplifiers employ a correlation function between the measured signal and the reference signal, which is an integration over 300 ms windows that is repeated over the 80-second stimulation window for averaging. The complex result of this autocorrelation is representative of the amplitude and phase of the measured signal. In total, eight different frequencies were used: 250 Hz, 500 Hz, 750 Hz, 1 kHz, 2 kHz, 3 kHz, 4 kHz, and 6 kHz. The stimulation amplitude varied across the different frequencies to obtain a sound level of approximately 60 dB HL, corresponding to a clinically relevant stimulation level. A commercial Baha 5 SuperPower sound processor was coupled to a TU-1000 skull simulator (Nobelpharma Inc., Göteborg, SE) [33] using an abutment and BI300 adapter to determine the force level corresponding to 60 dB HL as defined in the manufacturer's fitting software. In a second step, a Baha 5 SuperPower actuator, i.e., without sound processor electronics, was used to obtain the same force levels. The corresponding stimulation voltages were used in all cadaver measurements. In addition to a Baha 5 SuperPower actuator, an Osia OSI200 actuator was used in a similar calibration by coupling directly to the BI300 adapter.

During each single-frequency stimulation step, four lock-in amplifiers were used to acquire data. Three were connected to a sensor input, being either the LDV signal or one of two pressure probes. The fourth lock-in amplifier was connected to both pressure probes to be able to calculate the differential signal between the two. The applied stimulation signal was fed back to each of the lock-in amplifiers as a reference signal. Data was recorded using a custom Matlab interface (MathWorks, Massachusetts, USA).

### 2.3. Experimental Procedure

#### 2.3.1. Feasibility for Surgical Simplification of the OSI Implant (*N* = 2)

For one of the specimens, Head 16, an additional step was added at the start of the experimental procedure to assess the possibility of simplifying the surgical procedure. This step was added before implantation of the pressure sensors to avoid having to change the surgical setting once these fragile parts are implanted. It consisted of placing the OSI200 actuator onto the skull directly next to position 1 before the placement of a bone screw. The skin and periosteal pocket were closed with sutures, and the actuator was then stimulated, and the vibrational response of the cochlear promontory was recorded. In a second step, modeling clay (Play-Doh, Hasbro, Pawtucket, USA) was used to simulate a more rigid connection to the bone. The modeling clay is considered to be a rudimentary representative for tissue undergrowth after a foreign body reaction. The obtained data was saved for comparison with other datasets obtained during the complete experiment. The implant was then removed before continuing with the remainder of the experiment. To strengthen the findings of these measurements, this feasibility has been repeated in a different sample to obtain a sample size of 2. In this repeated experiment, the experimental step with the modeling clay was omitted.

#### 2.3.2. Experimental Procedure

In this step, three BI300 bone screws were implanted following the manufacturer's surgical guidelines on the three previously specified anatomical locations. To simulate the effect of osseointegration and to provide a more stable coupling, dental cement (Dyract Seal, Dentsply Sirona, Pennsylvania, USA) was used around the BI300 bone screw and the skull. A Bone Bed Indicator was used to identify any interferences between the bone and the implant as prescribed in the surgical instructions and outlined by Goldstein et al. [34]. In case interference was detected between the Indicator and the bone, additional bone polishing was performed to ensure clearance between the components.

The middle ear was then submerged in saline and a cochleostomy was carefully performed into the scala vestibuli by blue lining with a 0.5 mm diamond skeeter bur and opening with a 0.35 mm manual perforator. Subsequently, the tip of an FOP-M260 fiber-optic pressure sensor (FISO Technologies Inc., Quebec, CA) was inserted approximately 200 *μ*m using micromanipulators and sealed with a minimal amount of dental alginate (dental impression material; Alginoplast1, Heraeus Kulzer GmBH, DE). The same procedure was repeated for the scala tympani. Afterward, the excess saline was slowly removed, and a control measurement was performed to detect any changes in cochlear impedance reflected in the METF due to the sensor implantation. In general, the technique of intracochlear pressure measurements that is followed in this work has been documented by Borgers et al. The technique outlined in this work has been based on prior work by other authors [12, 13, 21]. Similar to Borgers et al. [23], the middle ear was submerged in saline again before dental cement was applied on the bone surrounding the cochleostomy to fix the sensors in place. After cementing each sensor, it was removed from the micromanipulators. A third control measurement was performed after removing the excess saline to check the complete functionality of the setup before proceeding with the remainder of the procedure. Due to the long experimental duration ranging between 7 and 8 hours, the middle and inner ear was wetted with saline every hour to preserve the specimen quality.

The OSI200 actuator was then placed at position 2 and stimulated at 60 dB HL after suturing the periosteal pocket and overlaying skin. The intracochlear pressure and promontory velocities were measured simultaneously. Test-retest variability was checked by repeating the same stimulation sequence immediately after. Next, the actuator was moved to position 3 where this procedure was repeated. Afterward, the OSI200 actuator was replaced by a Baha 5 SuperPower actuator mounted on an abutment. Stimulation was also performed at 60 dB HL. The Baha 5 SuperPower actuator and abutment were removed afterward, and an OSI200 actuator was placed at location one for stimulation at 60 dB HL. Similarly, the periosteal pocket and skin were sutured before measuring the ICP and promontory velocities for positions 2 and 3.

The effect of reactive bone undergrowth is finally simulated in a two-step approach using orthopedic bone cement (Palacos Fast R+G; Heraeus Medical GmbH, Wertheim DE). First, bone cement was applied under half the footprint of the implant to simulate uneven reactive bone undergrowth. After measuring the promontory velocity and intracochlear pressure as a response to 60 dB HL stimulation, the second half of the device footprint was underfilled with cement and the measurements were repeated.

### 2.4. Data Analysis

Raw data were imported into Matlab for further processing. Preprocessing was performed on the raw complex data before conversion to pressure or velocity. Datapoints with a signal-to-noise ratio lower than 1 were omitted. The noise floor for both pressure sensor channels was determined by submerging the sensor in water and measuring the sensor response without stimulation.

During data processing, it was noted that the stimulation level of the OSI200 actuator at 6 kHz was a factor 10 too low during the experiment. In a verification experiment afterward, it was verified that the promontory velocity can be linearly scaled. Results presented in the following section, therefore, show corrected promontory velocities at 6 kHz. Intracochlear pressure data could not be corrected.

After preprocessing data was further analyzed in Matlab with raw signals converted to pressure and/or velocity. The frequency-dependent amplitude and phase response of the pressure and velocity signals were derived by calculating the magnitude and phase of the complex response, respectively. Different test conditions were compared using a paired *t*-test with a Bonferroni correction for multiple comparisons (*n* = 8 frequencies). This test was selected to make intraspecimen comparisons between either ICP or promontory velocities measured at the different positions. The low sample size used (*N* = 4) inhibits generalizing the obtained results to the population level. Valuable intraspecimen comparisons are however possible using the obtained data. Normality of the data was checked with a Shapiro-Wilk normality test. Error bars were defined as 1.96 times the variance of the average amplitude or phase response.

To investigate the relationship between the implant location and either the promontory velocity or the differential pressure, the Spearman correlation was determined. Data measured at location 3 with either the OSI200 or Baha actuator was pooled as both devices stimulated at the same intensity.

## 3. Results

### 3.1. Efficiency in Power Transfer with respect to Implant Location

**Figure 3:**
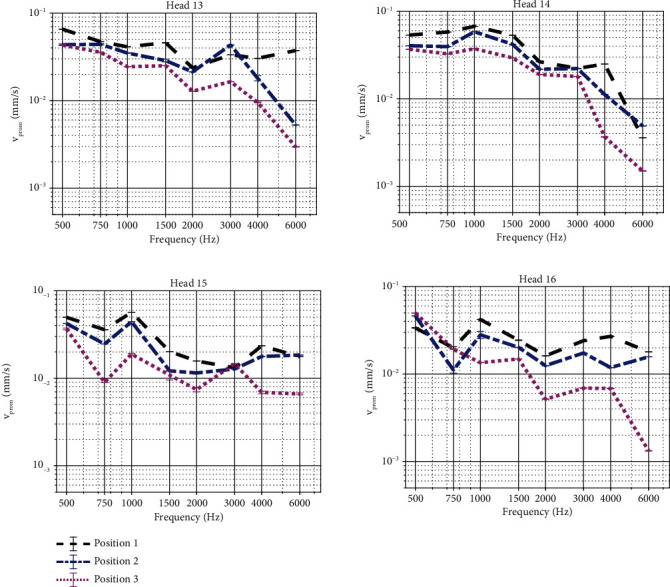
Velocity of the cochlear promontory when stimulating the skull with an actuator coupled at different anatomical locations. Results obtained with an OSI200 actuator are shown in black, blue, and pink for positions 1, 2, and 3, respectively. Magnitudes are plotted together with error bars. The response at positions 2 and 3 differs significantly (*p* < 0.0001) from each other at all frequencies.

**Figure 4:**
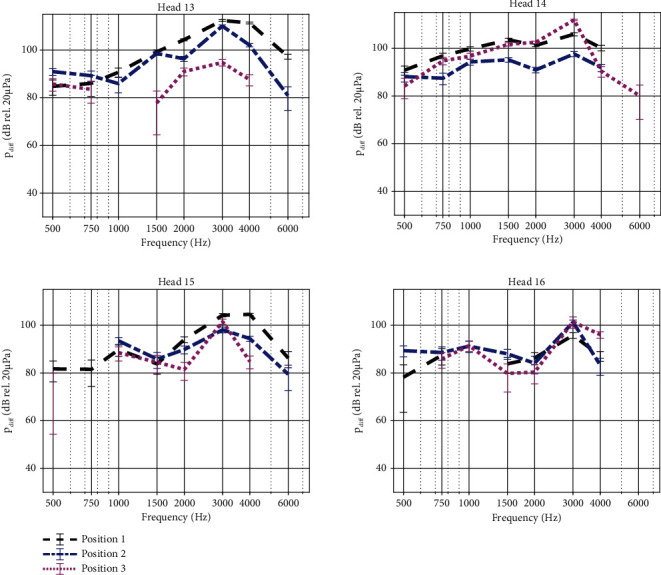
Intracochlear pressure difference when stimulating the skull with an actuator coupled at different anatomical locations. Results obtained with an OSI200 actuator are shown in black, blue, and pink for positions 1, 2, and 3, respectively. Magnitudes are plotted together with error bars. The response at positions 2 and 3 differs significantly (*p* < 0.05) from the response at position 1 other at most frequencies. Exceptions in the comparison between positions 1 and 2 include 0.75 kHz for Heads 13 (*p* = 0.48), 15 (*p* = 0.68), and 16 (*p* = 1); 2 kHz for Head 15 (*p* = 0.39); and 3 kHz for Head 16 (*p* = 1). Exceptions in the comparison between positions 1 and 3 include 0.5 kHz for Head 13 (*p* = 0.52) and 1-2 kHz for Head 15 (*p* = 0.099 and *p* = 1, respectively).

Measurements of the promontory velocity for each specimen show that the vibration amplitude increases when stimulating closer to the cochlea (full statistics in Tables S1 and S2, Figure 3). Overall, the relative differences between subsequent positions, i.e. position 1 vs. position 2 and position 2 vs. position 3, show an increase in vibrational response up to 8 dB for frequencies up to 4 kHz and an increase up to 20 dB for higher frequencies. Exceptions to this general trend are observed at 3 kHz, where vibration amplitudes are similar (Heads 14 and 15) or higher (Head 13) further away from the cochlea and at frequencies below 1 kHz where vibration amplitudes are also often higher further away from the cochlea. Trends in intracochlear pressure differences (Tables S3 and S4, Figure 4) are less clear, showing more subtle differences between the different test locations. For all specimens, the phase lag of the promontory velocity increases approximately by 500° per decade while for the intracochlear pressure difference this increase is less with approximately 400° per decade. Only Head 16 diverts from this trend, showing an increase in phase lag up to 1000° per decade for the intracochlear pressure difference. Phase plots have been provided in Figures S2 and S3 as no phase differences were observed between the different test conditions per specimen.

**Figure 5:**
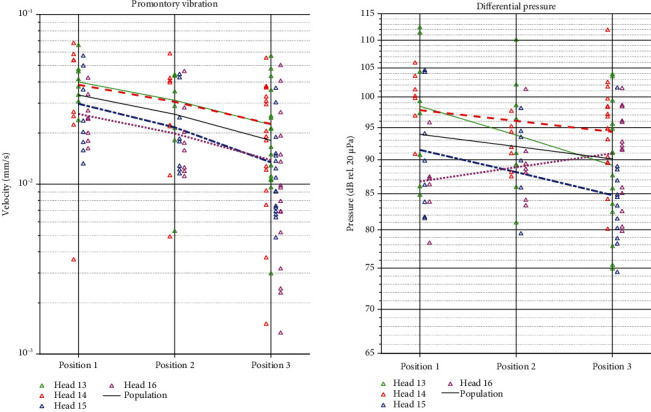
Promontory velocity (a) and differential pressure (b) measured at the different experimental locations for the four specimens. Measurement points are depicted by markers while a linear fit is depicted in a solid line. A linear fit to the data of all specimens is depicted in black.

Promontory velocities measured at the different locations for the four specimens are depicted in Figure 5(a), with the measurement points indicated with markers and a linear fit using a solid line. A linear fit was made for the measurement data grouped over all specimens and is depicted in black. The Spearman correlation coefficients are provided in Table 1, indicating that for the promontory velocity a statistically significant correlation where the velocity decreases with the increasing distance between implant and cochlea could be established in three out of the four specimens and on the group level (*p* < 0.01). Only in Head 14, the negative correlation is not significant (*p* = 0.059).

Figure 5(b) illustrates the case for the differential pressure, showing a negative trend in three out of the four specimens. Only in Head 16, a positive trend was found. When looking at the correlation coefficients, no significant trends could be observed.

### 3.2. Comparison between the Actuators of the Osia OSI200 and a Percutaneous Device at Their Intended Surgical Locations

**Figure 6:**
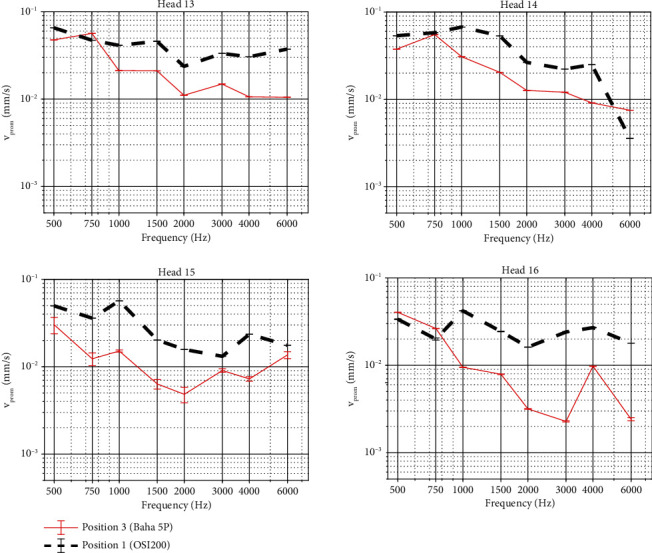
Velocity of the cochlear promontory when stimulating the skull with a Baha 5 Power actuator (red) or an Osia OSI200 actuator (black) stimulating at 60 dB HL. Magnitudes are plotted together with error bars. The responses between the different curves differ significantly (*p* < 0.0001) from each other at all frequencies.

Comparison of the promontory velocity and the intracochlear pressure difference, when either stimulating the specimen with a Baha 5 SuperPower actuator or an Osia OSI200 actuator at their intended anatomical locations, yields higher amplitudes for the OSI200 actuator. Since the output force of both actuators is calibrated to the same output force level, these differences illustrate mainly the effect of different distances to the cochlea. For completeness, the promontory velocity measured when both devices are mounted on the same location is provided in Figure S1, which takes into account a 3 dB measurement uncertainty on LDV measurements on the cochlear promontory. The measurement uncertainty of 3 dB was determined by comparing the test-retest variability of promontory measurements of subsequent measures in the same test condition. The promontory velocity is plotted for all specimens in Figure 6, each time showing the signal amplitude when stimulating with the Baha 5 SuperPower actuator in red and the amplitude when stimulating with the OSI200 actuator in black. For all specimens, the vibrational responses between both stimulation modalities differ significantly from each other (*p* < 0.0001, Table S5). In all but four cases, the promontory velocity is higher for the OSI200 actuator in position 1 compared to the Baha 5 SuperPower actuator in position 3. Promontory vibrations can differ up to 20 dB between the two test conditions and are in general larger for stimulation frequencies above 1 kHz. At stimulation frequencies of 500 Hz and 750 Hz for head 16, 750 Hz for head 13, and 6 kHz for head 14, vibration amplitudes are higher for the Baha 5 SuperPower actuator in position 3 compared to the OSI200 actuator in position 1.

**Figure 7:**
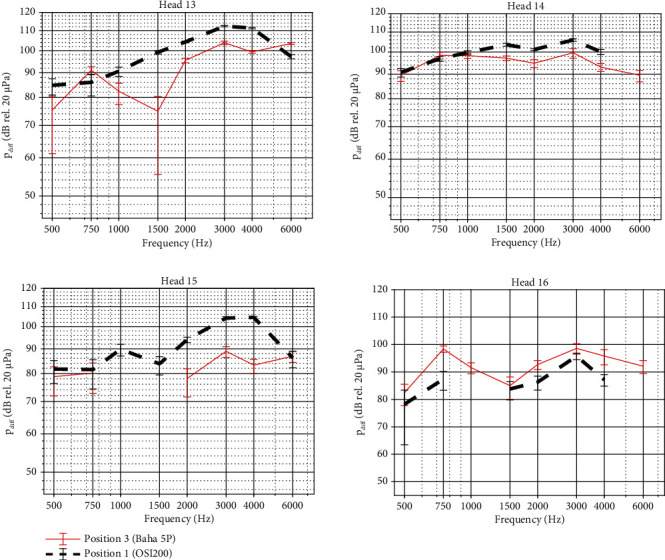
Intracochlear pressure difference when stimulating the skull with a Baha 5 Power actuator (red) or an Osia OSI200 actuator (black) stimulating at 60 dB HL. Results are shown for the four specimens. Magnitudes are plotted together with error bars. The responses between the different curves differ significantly from each other at most frequencies (*p* < 0.01). Exceptions are observed at 0.5 kHz for Heads 14 and 16 (*p* = 1).

Similar findings can be observed for the intracochlear pressure difference shown in Figure 7. For head 16, however, the differential pressure is significantly larger for the Baha 5 SuperPower actuator at position 3 at all frequencies (*p* < 0.01, Table S6) except for 500 Hz, where both test conditions give similar pressure amplitudes (*p* = 1). For the three other specimens, the differential pressure is significantly larger for OSI200 (*p* < 0.01, Table S6) except for 500 Hz in head 14 (*p* = 1).

For both the promontory velocity and the intracochlear pressure differences, the phase lags are similar to those mentioned in the prior paragraph with 500° and 400° per decade, respectively. Phase plots have been provided in Figures S4 and S5 as no phase differences were observed between the different test conditions per specimen.

### 3.3. Impact of Cortical Bone Undergrowth on OSI Performance

**Figure 8:**
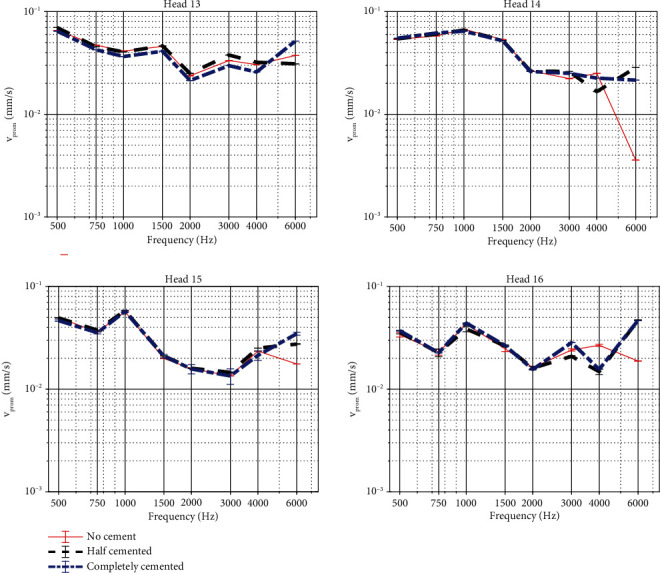
Velocity of the cochlear promontory when stimulating the skull with an actuator coupled with a BI300 bone screw. Results are shown for the four specimens, with different colors illustrating different test cases to simulate reactive bone undergrowth. Magnitudes are plotted together with error bars. The response when partially or completely cemented differ significantly from the no cement condition at most frequencies (*p* < 0.0001). Exceptions in the comparison between partial and no cement are observed for Head 16 at 0.75 and 3 kHz (*p* = 1). Exceptions in the comparison between complete and no cement are observed for Head 15 at 1 and 3 kHz (*p* = 0.13 and *p* = 0.74, respectively) and for Head 16 at 0.75 and 2 kHz (*p* = 0.24 and *p* = 0.11, respectively).

The promontory velocities measured when simulating reactive tissue undergrowth are shown in Figure 8 for the four specimens. It can be seen that the vibrational responses mostly overlap and that relative differences between the test conditions differ only up to 3 dB for frequencies up to 4 kHz. At 6 kHz, larger differences up to 16 dB can be observed. To estimate the importance of these differences, the test-retest variability of promontory measurements was assessed by examining differences in promontory velocity between repeated measures in the same condition. By analyzing these datasets, the test-retest variability turned out to be ±3 dB (6.5 *μ*m/s).

**Figure 9:**
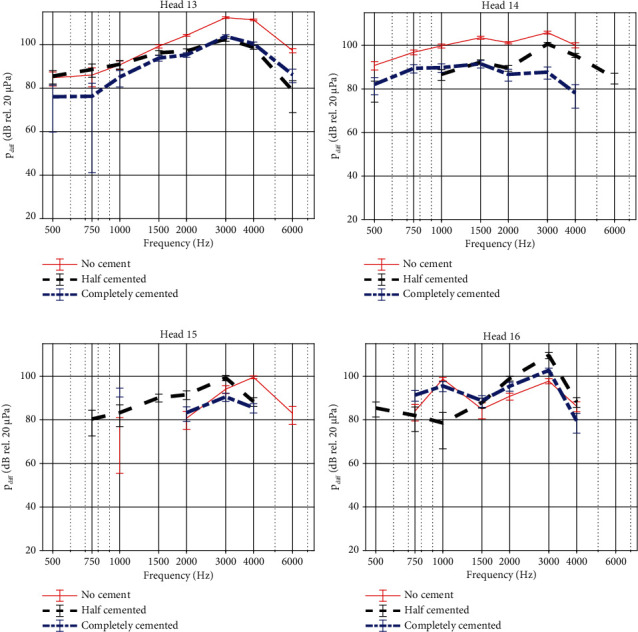
Intracochlear pressure difference when stimulating the skull with an actuator coupled with a BI300 bone screw. Results are shown for the four specimens, with different colors illustrating different test cases to simulate reactive bone undergrowth. Magnitudes are plotted together with error bars. The response when partially or completely cemented differs significantly from the no cement condition at most frequencies (*p* < 0.0001). Exceptions in the comparison between partial and no cement are observed for Head 13 at frequencies below 1 kHz (*p* = 1 for 0.5 and 1 kHz and *p* = 0.2 for 0.75 kHz), for Head 15 at 0.75 kHz (*p* = 0.24), and for Head 16 at 0.5 kHz (*p* = 1). Exceptions in the comparison between complete and no cement are observed for Head 15 at 0.5 kHz (*p* = 1).

Similar phenomena are observed for the intracochlear pressure differences (Figure 9), showing only subtle differences between the different test cases. Only in Head 14, a clear reduction in sound pressure is observed when bone cement is added.

For both promontory velocity and intracochlear pressure differences, phase lags between the different test conditions are nearly identical. Only marginal differences can be observed. Phase plots have been provided in Figures S6 and S7 as no phase differences were observed between the different test conditions per specimen. The complete statistical information for these variables is collected in Tables S7-10.

### 3.4. Importance of the Implant Fixation System

**Figure 10:**
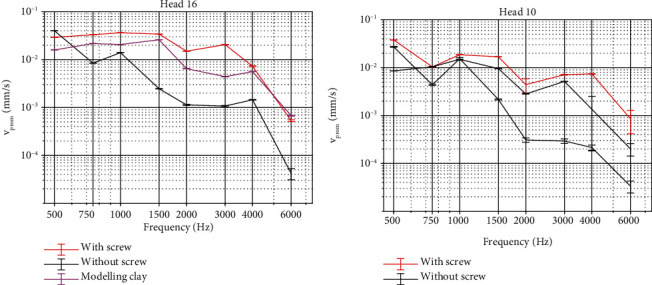
Velocity of the cochlear promontory when stimulating the skull with an actuator coupled with a BI300 bone screw without using this screw. (a) Measurements obtained with H16. (b) Measurements obtained with H10. Magnitudes are plotted together with error bars. The responses between the different curves significantly from each other at all frequencies (*p* < 0.01).

As the feasibility experiments for surgical simplification were performed before implantation of the fiber-optic pressure probes, results focus only on the vibration of the cochlear promontory in response to the different test conditions.  Figure 10 depicts results that on the left panel data is shown from the first set of experiments with Head 16 and on the right panel data is shown for Head 10. Both panels of the figure show a significant reduction (*p* < 0.01, Table S11) in vibration amplitude over all frequencies when the OSI200 actuator is not rigidly coupled to the skull using the BI300 bone implant. When placing the actuator on the skull without the bone implant but using modeling clay to improve the coupling stability the vibration amplitude remains significantly lower compared to when the device is placed on the BI300 bone implant (*p* < 0.0001, Table S12). A relative decrease up to 30 dB is found in the promontory velocity when the actuator is not mounted on a bone screw. Moreover, the datasets differ by up to 20 dB where the actuator is not mounted on the skull. When modeling clay is used to create a more rigid coupling to the skull, relative differences are reduced to between 2 and 6 dB for most frequencies. Only at 3 kHz, a large difference of 12 dB remains. All test conditions show an increasing phase delay of approximately 500°/decade. Phase plots have been provided in Figure S8.

## 4. Discussion

### 4.1. Efficiency in Power Transfer with respect to Implant Location

The first aim of the study was to investigate if implanting the OSI200 actuator closer to the ear canal is beneficial for the stimulation efficiency overall and for different frequencies. Results obtained in this study showed a significant difference between the different anatomical positions in terms of promontory vibration (*p* < 0.0001) and differential intracochlear pressure (*p* < 0.05) and a significant correlation between the implant location and the transmitted power in terms of promontory vibration (*p* < 0.01) for all but one specimen. Overall, promontory velocity data showed higher responses at position 1 compared to positions 2 and 3. Vibration magnitudes below 1000 Hz are expected to be similar as the skull is assumed to move as a rigid body at these frequencies [6, 8, 35]. At higher frequencies, an increase up to 20 dB was found between the different positions, which can be attributed to the reduced distance to the cochlea. Based on the study of Stenfelt and Goode, a transmission loss of 0.5-1.5 dB per cm away from the cochlea can be expected [10]. In the current study, higher relative differences were found between positions, both for promontory velocity data and for ICP. The movement of the specimen at different frequencies is constrained by the method for fixating the head specimen to the vibration insulation table. An effect of the fixation method would mainly be expected at lower frequencies, where the skull is assumed to move as a rigid body as mainly lateral motion of the skull is different than an in vivo situation due to the fixation method. The exact effect of the specimen fixation to the table was not quantified but is expected not to influence the outcomes of the relative comparisons made in this study above 1 kHz. The findings reported in this work are however consistent with the findings of other authors [8, 9], including a study on the impact of OSI stimulation location on the promontory velocity [6]. Secondly, a significant correlation was found between the promontory vibration and the actuator position with respect to the ear canal in three out of the four specimens and on the group level (*p* < 0.01), building on the hypothesis that the stimulation location is of importance in terms of transferring energy towards the cochlea. Only when stimulating at a frequency of 3 kHz the trend of improved promontory velocity with decreased distance to the cochlea could not be observed. This is, however, consistent with the findings of Rohani and colleagues, who performed similar measurements for the Med-El™ BoneBridge® in human temporal bones [36]. Also for this device, an improvement in cochlear promontory vibration could be observed when comparing the distal retrosigmoid position with the more proximal transmastoid position. It should be noted that in this study no full heads were used, and not all bone conduction pathways are accounted for.

The present study adds to this knowledge base by the measurement of the intracochlear pressure difference. Despite technical challenges, qualitative data could be measured that confirm the findings obtained using promontory motion. The intracochlear pressure difference indicates a more complex mechanism that is involved in bone conduction hearing that is not captured when measuring the promontory vibration alone. More research is needed to disentangle the origins of these subtle differences at clinically relevant stimulation levels. The indications and trends identified by the performed experiments can however not be generalized to the entire population due to the limited sample size of *N* = 4. Future experiments should also include dynamic force measurements to allow measuring the applied stimulation frequency in each experimental step. That will allow comparing the obtained results more directly with other experimental findings as highlighted in the recent work of Prodanovic and Stenfelt [37]. In our work, we have employed relative measurements, which do not rely on absolute measurements of stimulation power.

### 4.2. Comparison between the Actuators of the Osia OSI200 and a Percutaneous Device at Their Intended Surgical Locations

The results of this study indicate a higher output performance of the OSI200 actuator compared to a standard percutaneous BC actuator when stimulated at 60 dB HL and implanted at their recommended positions, which is mainly shown for frequencies above 1 kHz. At lower frequencies, the performance of both devices was similar. As both actuators were matched in output force, corresponding to stimulation levels around 60 dB HL, the difference between both actuators is expected to be mainly determined by a different position. When both actuators would be compared on a system level (i.e., in vivo use), an additional gain on the high frequencies is expected, because the OSI200-actuator and Osia® Sound Processor are physically separated, reducing the risk for feedback and thus increasing the available gain at higher frequencies. The final stable gain will be patient-specific.

Recently, the first clinical studies have been published using this new system. In a recent study from Mylanus et al., a comparison between preoperative performance using a passive transcutaneous system (Baha BP110 on Softband) and postoperative outcomes with Osia showed significant improvement in speech perception and a reduction in aided hearing thresholds [38]. Similarly, Goycoolea et al. compared preoperative functional outcomes with a different passive transcutaneous device (Baha 5 Power on Softband) with postoperative results obtained with Osia. Results showed a higher functional gain of approximately 6 dB across frequencies. The difference is mainly dominated by the higher frequencies where a higher functional gain of more than 10 dB with Osia® was observed [5]. In both studies, the assumption was made, based on previous studies (e.g., [39]), that preoperative testing with a Softband might be a good indication for audiological performance with a passive transcutaneous BC device. In our study, the output performance of the Osia® OSI200 actuator was for the first time compared with those of the Baha® 5 SuperPower actuator in a percutaneous application where the actuators stimulated the specimen with an identical force level. A study by Huber and colleagues performed a similar experiment to compare the output of the percutaneous Cochlear™ Baha® BP110 with the active transcutaneous Med-El™ BoneBridge® [40]. Stimulation however happened on a system level, by fitting the device's sound processor with the maximum power output level (MPO) and presenting an acoustic stimulus to the specimen. This different stimulation modality impedes comparing both studies.

Similar observations can be made for a study describing a comparison between the Ponto 3 percutaneous and Sentio active transcutaneous BCI by Ghoncheh and colleagues [41]. A cadaver study is presented where both devices are compared with respect to the promontory vibration generated when stimulating both devices with an output force corresponding to a 90 dB SPL input level. As this is a system-level parameter, it is difficult to estimate actuator-related aspects impeding a comparison with the current study.

### 4.3. Impact of Cortical Bone Undergrowth on OSI Performance

Bone cement was used to mimic reactive bone undergrowth. Bone undergrowth or remodeling might occur after flattening the skull and is dependent on the amount of motion of the implant [42]. In some cases, reactive bone growth can occur under the implant, but to the authors' knowledge, it is not known whether this might alter sound transmission. The different cementing conditions did not differ concerning the promontory velocity, indicating that reactive bone growth over time would not alter the output performance of the device. In the differential pressure, some differences are however observed in the data. A higher differential pressure when no cement is applied in three of the four heads indicates that there could be a subtle contributor in the sound conduction pathway that is not captured in the promontory velocity.

### 4.4. Importance of the Fixation System

The use of a bone screw to rigidly fix an actuator to the bone of a recipient has been debated [24]. Although a nonrigid coupling might be feasible in pure conductive hearing loss cases where little amplification is needed, significantly more power can be transmitted when firmly coupled to the skull. The feasibility study performed (*N* = 2) indicates that rigid fixation of the actuator to the skull is key in providing efficient and consistent BC stimulation. Power transfer to the cochlea is reduced by up to 30 dB while test-retest variability indicates large variations in output power when a nonrigid coupling is used. Despite this being an ex vivo study, the results provide more insights into the importance of a rigid implant coupling to ensure optimal stimulation. As tissue under/overgrowth after implantation might provide additional stiffness over time, modeling clay was used to simulate the effect of this foreign body reaction. This led to more power reaching the cochlea, yet there are still important reductions in power with respect to the rigid coupling method.

These results underline the importance of proper implant fixation, showing that a nonrigid fixation provides suboptimal results. Optimizations of power transfer to the cochlea could be optimized with rigid fixation, by changing the fixation method itself [43, 44].

## 5. Conclusion

A novel active bone conduction actuator was characterized by measuring the velocity of the cochlear promontory in four human cadaveric heads, supplemented by the measurement of the intracochlear pressure difference. The bone conduction sensitivity at the cochlear level was found to be optimal with an actuator attachment closer to the cochlea (Osia position) as compared to a more distant position (Baha position) when stimulating with the same mechanical vibration, especially at the higher frequencies. Preliminary results show that the effect of (simulated) bone under the implant does not alter the device output but that the use of rigid fixation of the device to the skull is key to obtaining a stable and optimal output performance of the device.

## Figures and Tables

**Table 1:** The Spearman correlation for the different specimens individually and on a group level.

Specimen	Correlation coefficient for promontory velocity (*p* value)	Correlation coefficient for differential pressure (*p* value)
Head 13	-0.46 (0.0079)	-0.34 (0.06)
Head 14	-0.34 (0.059)	-0.21 (0.27)
Head 15	-0.58 (0.00056)	-0.38 (0.055)
Head 16	-0.53 (0.0017)	0.27 (0.16)
Group	-0.43 (5.1.10^−7^)	-0.16 (0.096)

## Data Availability

The reported data used to support the findings of this study are available from the corresponding author upon reasonable request.
