# Supplementary material for: The Impact of Location and Device Coupling on the Performance of the Osia System Actuator
Source: Biomed Res Int. 2022 Apr 2;2022:9079903. doi: 10.1155/2022/9079903 (PMC8994691; doi:10.1155/2022/9079903)
Supplement: Supplementary Materials — The complete statistical results are provided as Supplementary Materials to this work. [file 9079903.f1.docx]

## Supplementary material – Stimulation intensity with both devices at Position 3


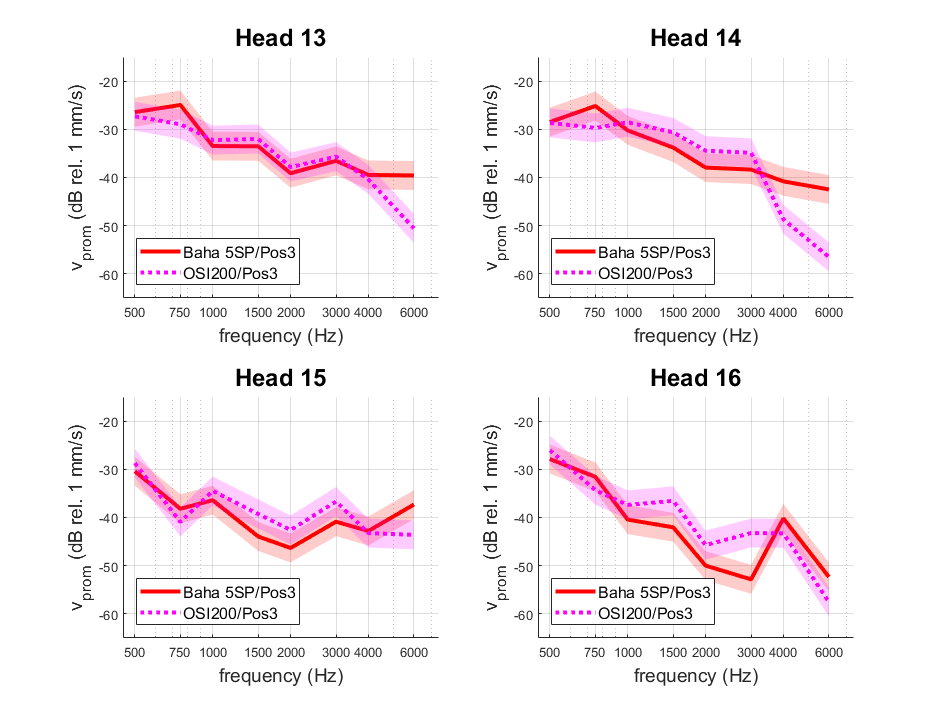


Figure S. 1: Stimulation intensity at the cochlear promontory when using both devices to stimulate at 60 dB HL at position 3. The measured promontory vibration is shown in a solid line for the Baha 5 SuperPower actuator (red) and the OSI 200 actuator (pink). Measurement uncertainty is indicated for each measurement using a shaded region.

## Supplementary material – Phase plots for all test cases


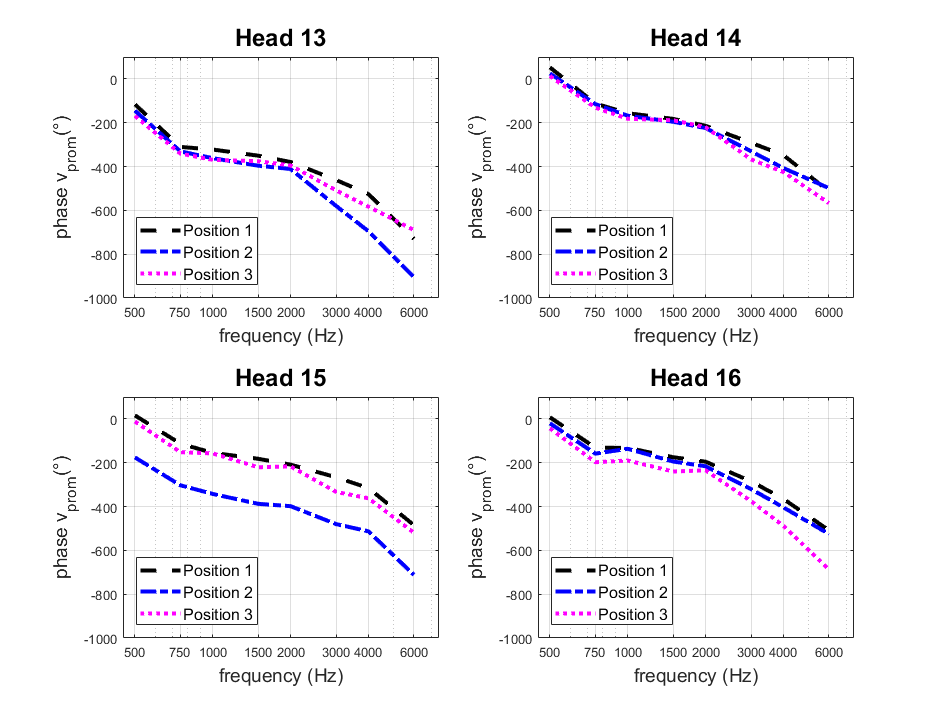


Figure S. 2: Phase of the cochlear promontory vibation when stimulating the skull with an actuator coupled at different anatomical locations. Results obtained with an OSI200 actuator are shown in black, blue, and pink for positions 1, 2, and 3, respectively


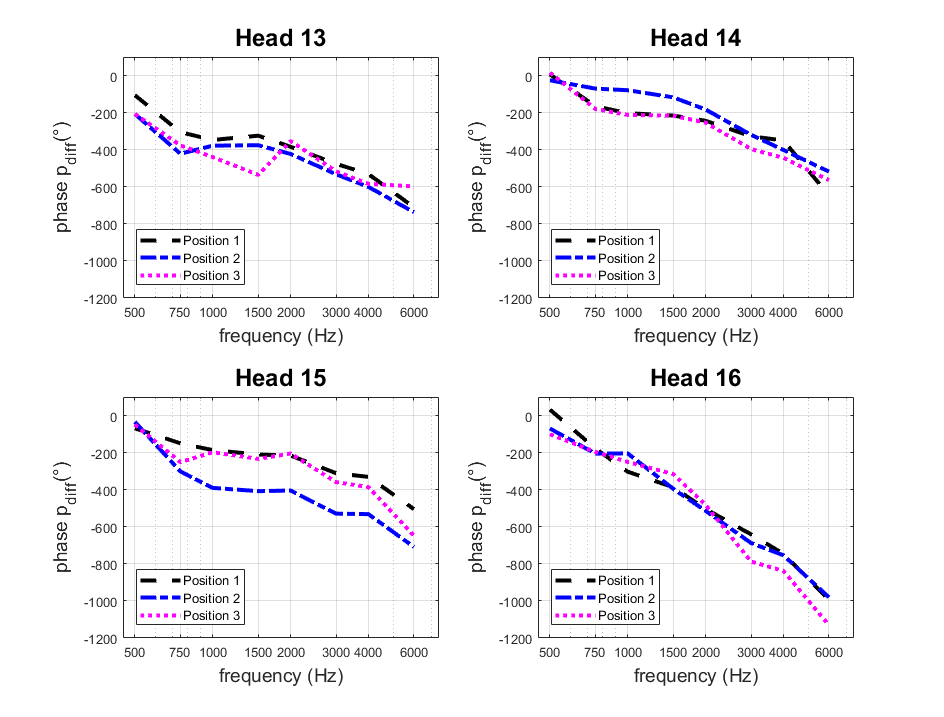


Figure S. 3: Phase of the intracochlear pressure difference when stimulating the skull with an actuator coupled at different anatomical locations. Results obtained with an OSI200 actuator are shown in black, blue, and pink for positions 1, 2, and 3, respectively.


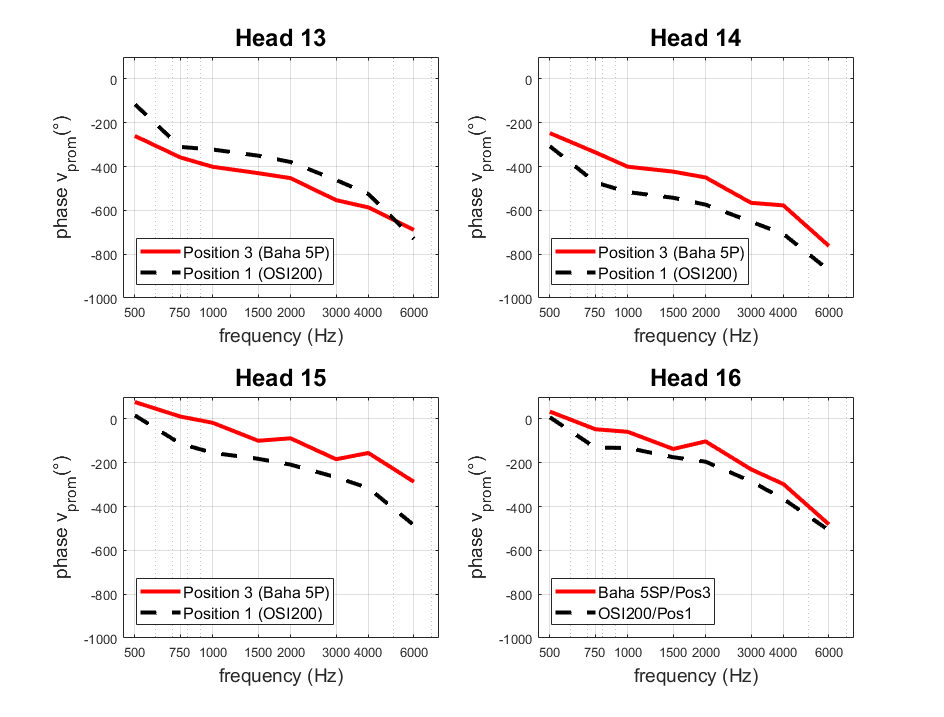


Figure S. 4: Phase of the cochlear promontory vibration when stimulating the skull with a Baha 5 Power actuator (red) or an Osia OSI200 actuator (black) stimulating at 60 dB HL.


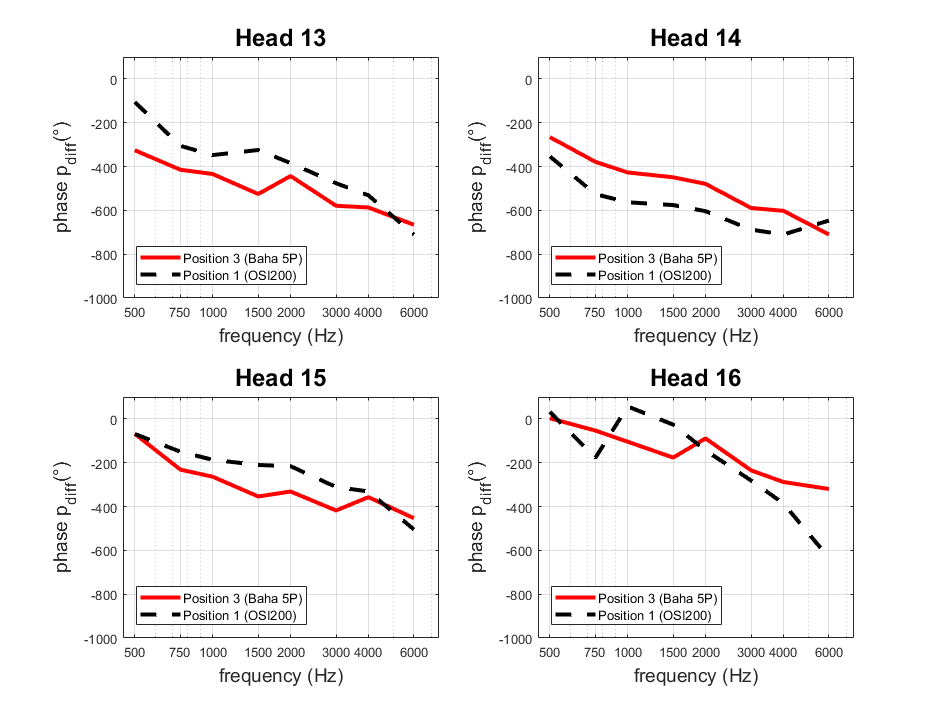


Figure S. 5: Phase of the intracochlear pressure difference when stimulating the skull with a Baha 5 Power actuator (red) or an Osia OSI200 actuator (black) stimulating at 60 dB HL.


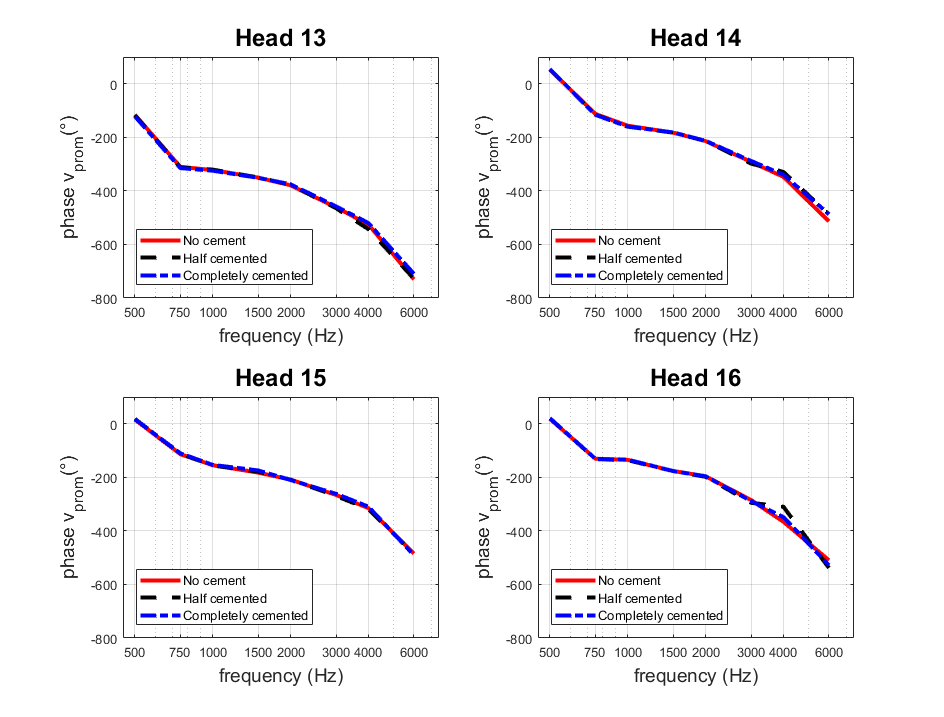


Figure S. 6: Phase of the cochlear promontory vibration when stimulating the skull with an actuator coupled with a BI300 bone screw. Results are shown for the four specimens, with different colours illustrating different test cases to simulate reactive bone undergrowth


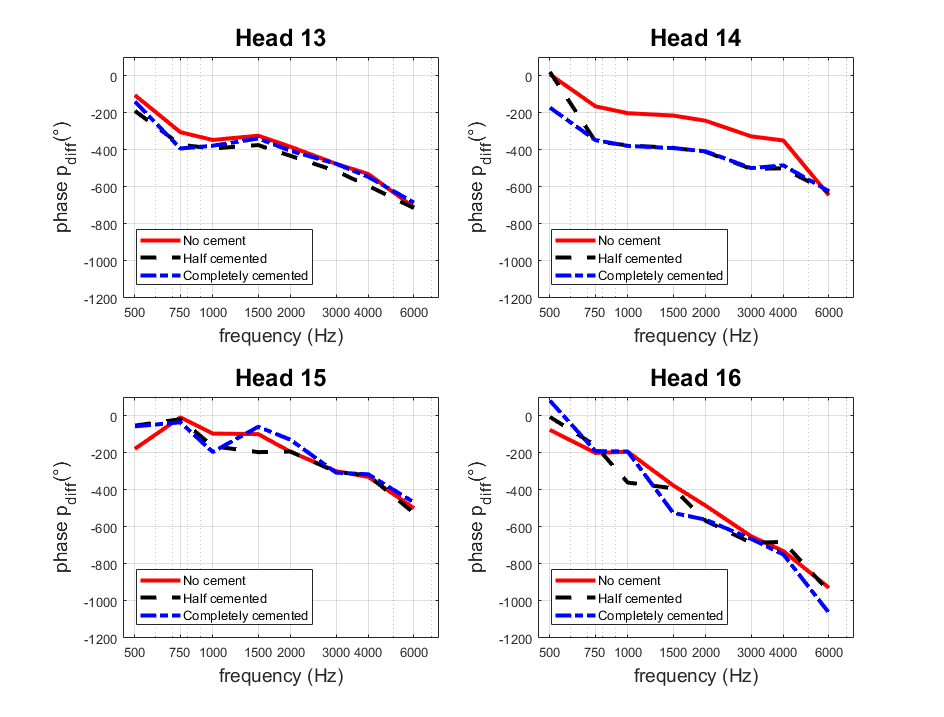


Figure S. 7: Phase of the intracochlear pressure difference when stimulating the skull with an actuator coupled with a BI300 bone screw. Results are shown for the four specimens, with different colours illustrating different test cases to simulate reactive bone undergrowth


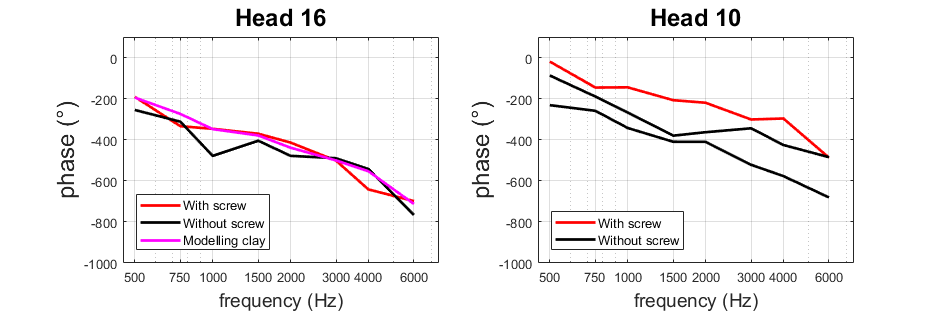


Figure S. 8: Phase of the cochlear promontory when stimulating the skull with an actuator coupled with a BI300 bone screw without using this screw. Left: Measurements obtained with H16. Right: Measurements obtained with H10.

## Supplementary material – Statistical analyses

### Anatomical location - Promontory velocity

Table S. 1: Comparison of promontory velocity between OSI200 at position 1 versus position 2. Data is shown as mean ± standard deviation. P-values are Bonferroni-corrected for multiple comparisons. P-values that are >1 because of the Bonferroni correction are capped to a value of 1, while p-values lower than 0.0001 are capped to 0.0001 for readability purposes.

|  | **Frequency→**  **Dataset ↓** | **0.5 kHz** | **0.75 kHz** | **1 kHz** | **2 kHz** | **3 kHz** | **4 kHz** | **6 kHz** |
| --- | --- | --- | --- | --- | --- | --- | --- | --- |
| **Head 13** | **OSI200 pos. 1** | 0.066 ± 0.00023 (N=288) | 0.047 ± 0.00011 (N=255) | 0.041 ± 0.00033 (N=228) | 0.046 ± 0.00061 (N=210) | 0.024 ± 3.6e-05 (N=193) | 0.033 ± 3.4e-05 (N=180) | 0.031 ± 6.4e-05 (N=168) |
|  | **OSI200 pos. 2** | 0.044 ± 0.0019 (N=286) | 0.044 ± 0.0014 (N=256) | 0.035 ± 0.00067 (N=225) | 0.029 ± 0.002 (N=209) | 0.021 ± 0.00018 (N=194) | 0.043 ± 0.00024 (N=178) | 0.018 ± 0.0024 (N=165) |
|  | **p** | <0.0001 | <0.0001 | <0.0001 | <0.0001 | <0.0001 | <0.0001 | <0.0001 |
| **Head 14** | **OSI200 pos. 1** | 0.054 ± 0.0001 (N=209) | 0.058 ± 0.00013 (N=192) | 0.067 ± 0.00015 (N=173) | 0.053 ± 3.2e-05 (N=158) | 0.027 ± 9.7e-06 (N=150) | 0.022 ± 9e-06 (N=136) | 0.025 ± 5.7e-06 (N=129) |
|  | **OSI200 pos. 2** | 0.04 ± 4.1e-05 (N=209) | 0.04 ± 2.6e-05 (N=188) | 0.058 ± 2.4e-05 (N=171) | 0.042 ± 1.5e-05 (N=158) | 0.022 ± 2.1e-05 (N=146) | 0.022 ± 3.2e-05 (N=137) | 0.011 ± 1.7e-05 (N=123) |
|  | **p** | <0.0001 | <0.0001 | <0.0001 | <0.0001 | <0.0001 | <0.0001 | <0.0001 |
| **Head 15** | **OSI200 pos. 1** | 0.05 ± 0.00024 (N=289) | 0.036 ± 1.8e-05 (N=255) | 0.057 ± 1.5e-05 (N=229) | 0.02 ± 1.5e-05 (N=208) | 0.016 ± 1.2e-05 (N=191) | 0.013 ± 1.3e-05 (N=179) | 0.024 ± 1.9e-05 (N=166) |
|  | **OSI200 pos. 2** | 0.042 ± 3.8e-05 (N=286) | 0.025 ± 1.5e-05 (N=254) | 0.044 ± 1.4e-05 (N=228) | 0.012 ± 1.1e-05 (N=207) | 0.011 ± 1.2e-05 (N=190) | 0.013 ± 1.5e-05 (N=176) | 0.018 ± 2.3e-05 (N=167) |
|  | **p** | <0.0001 | <0.0001 | <0.0001 | <0.0001 | <0.0001 | <0.0001 | <0.0001 |
| **Head 16** | **OSI200 pos. 1** | 0.034 ± 0.00063 (N=287) | 0.02 ± 0.0012 (N=252) | 0.042 ± 0.00011 (N=227) | 0.024 ± 0.00034 (N=207) | 0.016 ± 0.00016 (N=190) | 0.024 ± 0.00066 (N=177) | 0.027 ± 0.00021 (N=166) |
|  | **OSI200 pos. 2** | 0.046 ± 0.00018 (N=282) | 0.011 ± 0.0016 (N=252) | 0.029 ± 0.0054 (N=226) | 0.02 ± 0.00014 (N=206) | 0.012 ± 9e-05 (N=189) | 0.017 ± 9.6e-05 (N=179) | 0.012 ± 2.4e-17 (N=165) |
|  | **p** | <0.0001 | <0.0001 | <0.0001 | <0.0001 | <0.0001 | <0.0001 | <0.0001 |

Table S. 2: Comparison of promontory velocity between OSI200 at position 1 versus position 3. Data is shown as mean ± standard deviation. P-values are Bonferroni-corrected for multiple comparisons. P-values that are >1 because of the Bonferroni correction are capped to a value of 1, while p-values lower than 0.0001 are capped to 0.0001 for readability purposes.

|  | **Frequency→**  **Dataset ↓** | **0.5 kHz** | **0.75 kHz** | **1 kHz** | **2 kHz** | **3 kHz** | **4 kHz** | **6 kHz** |
| --- | --- | --- | --- | --- | --- | --- | --- | --- |
| **Head 13** | **OSI200 pos. 1** | 0.066 ± 0.00023 (N=288) | 0.047 ± 0.00011 (N=255) | 0.041 ± 0.00033 (N=228) | 0.046 ± 0.00061 (N=210) | 0.024 ± 3.6e-05 (N=193) | 0.033 ± 3.4e-05 (N=180) | 0.031 ± 6.4e-05 (N=168) |
|  | **OSI200 pos. 3** | 0.043 ± 4.6e-05 (N=282) | 0.036 ± 0.0004 (N=254) | 0.024 ± 6.9e-05 (N=226) | 0.025 ± 3.6e-05 (N=207) | 0.013 ± 3.2e-05 (N=193) | 0.016 ± 3.1e-05 (N=178) | 0.0095 ± 0.00019 (N=166) |
|  | **p** | <0.0001 | <0.0001 | <0.0001 | <0.0001 | <0.0001 | <0.0001 | <0.0001 |
| **Head 14** | **OSI200 pos. 1** | 0.054 ± 0.0001 (N=209) | 0.058 ± 0.00013 (N=192) | 0.067 ± 0.00015 (N=173) | 0.053 ± 3.2e-05 (N=158) | 0.027 ± 9.7e-06 (N=150) | 0.022 ± 9e-06 (N=136) | 0.025 ± 5.7e-06 (N=129) |
|  | **OSI200 pos. 3** | 0.037 ± 2.4e-05 (N=129) | 0.033 ± 4.6e-05 (N=122) | 0.037 ± 3.2e-05 (N=111) | 0.029 ± 6.2e-05 (N=106) | 0.019 ± 0.00012 (N=98) | 0.018 ± 0.00011 (N=92) | 0.0037 ± 1.3e-05 (N=86) |
|  | **p** | <0.0001 | <0.0001 | <0.0001 | <0.0001 | <0.0001 | <0.0001 | <0.0001 |
| **Head 15** | **OSI200 pos. 1** | 0.05 ± 0.00024 (N=289) | 0.036 ± 1.8e-05 (N=255) | 0.057 ± 1.5e-05 (N=229) | 0.02 ± 1.5e-05 (N=208) | 0.016 ± 1.2e-05 (N=191) | 0.013 ± 1.3e-05 (N=179) | 0.024 ± 1.9e-05 (N=166) |
|  | **OSI200 pos. 3** | 0.037 ± 0.0016 (N=288) | 0.009 ± 3e-05 (N=255) | 0.019 ± 0.00087 (N=230) | 0.011 ± 0.0027 (N=207) | 0.0075 ± 0.00084 (N=190) | 0.015 ± 0.00022 (N=179) | 0.0069 ± 0.00045 (N=166) |
|  | **p** | <0.0001 | <0.0001 | <0.0001 | <0.0001 | <0.0001 | <0.0001 | <0.0001 |
| **Head 16** | **OSI200 pos. 1** | 0.034 ± 0.00063 (N=287) | 0.02 ± 0.0012 (N=252) | 0.042 ± 0.00011 (N=227) | 0.024 ± 0.00034 (N=207) | 0.016 ± 0.00016 (N=190) | 0.024 ± 0.00066 (N=177) | 0.027 ± 0.00021 (N=166) |
|  | **OSI200 pos. 3** | 0.05 ± 4.6e-05 (N=286) | 0.019 ± 4.4e-05 (N=254) | 0.014 ± 2e-05 (N=225) | 0.015 ± 6.4e-05 (N=206) | 0.0052 ± 1.8e-05 (N=189) | 0.0069 ± 0.00013 (N=178) | 0.0068 ± 8.5e-05 (N=165) |
|  | **p** | <0.0001 | <0.0001 | <0.0001 | <0.0001 | <0.0001 | <0.0001 | <0.0001 |

### Anatomical position - Differential pressure

Table S. 3: Comparison of differential intracochlear pressure between OSI200 at position 1 versus position 2. Data is shown as mean ± standard deviation. P-values are Bonferroni-corrected for multiple comparisons. P-values that are >1 because of the Bonferroni correction are capped to a value of 1, while p-values lower than 0.0001 are capped to 0.0001 for readability purposes.

|  | **Frequency→**  **Dataset ↓** | **0.5 kHz** | **0.75 kHz** | **1 kHz** | **2 kHz** | **3 kHz** | **4 kHz** | **6 kHz** |
| --- | --- | --- | --- | --- | --- | --- | --- | --- |
| **Head 13** | **OSI200 pos. 1** | 87 ± 3.9(N=274) | 89 ± 4.2(N=231) | 91 ± 3.1(N=228) | 99 ± 1.5(N=210) | 100 ± 0.83(N=193) | 110 ± 0.74(N=180) | 110 ± 0.74(N=168) |
|  | **OSI200 pos. 2** | 91 ± 2.6(N=286) | 90 ± 3.2(N=252) | 87 ± 3.8(N=211) | 99 ± 1.3(N=209) | 96 ± 1.9(N=194) | 110 ± 1(N=178) | 100 ± 1.4(N=165) |
|  | **p** | <0.0001 | 0,48 | <0.0001 | <0.0001 | <0.0001 | <0.0001 | <0.0001 |
| **Head 14** | **OSI200 pos. 1** | 91 ± 3.7(N=209) | 97 ± 2(N=192) | 100 ± 1.8(N=173) | 100 ± 1.4(N=158) | 100 ± 0.87(N=150) | 110 ± 1.3(N=136) | 100 ± 2.3(N=129) |
|  | **OSI200 pos. 2** | 88 ± 3.3(N=209) | 88 ± 3.5(N=187) | 94 ± 2.2(N=171) | 95 ± 1.3(N=158) | 91 ± 1.7(N=146) | 98 ± 2(N=137) | 92 ± 2.6(N=123) |
|  | **p** | <0.0001 | <0.0001 | <0.0001 | <0.0001 | <0.0001 | <0.0001 | <0.0001 |
| **Head 15** | **OSI200 pos. 1** | 84 ± 4.2(N=280) | 86 ± 3.8(N=230) | 90 ± 3.8(N=226) | 86 ± 3.6(N=202) | 94 ± 2.2(N=191) | 100 ± 1.3(N=179) | 100 ± 0.79(N=166) |
|  | **OSI200 pos. 2** | 83 ± 4.2(N=245) | 86 ± 4.4(N=235) | 93 ± 2.8(N=228) | 87 ± 4.1(N=196) | 90 ± 3(N=188) | 98 ± 2.4(N=176) | 94 ± 1.9(N=167) |
|  | **p** | 0,0032 | 0,68 | <0.0001 | 0,39 | <0.0001 | <0.0001 | <0.0001 |
| **Head 16** | **OSI200 pos. 1** | 85 ± 4.4(N=268) | 90 ± 4(N=237) | 85 ± 4(N=214) | 86 ± 3.6(N=200) | 87 ± 3.8(N=182) | 96 ± 2(N=177) | 88 ± 3.2(N=165) |
|  | **OSI200 pos. 2** | 90 ± 3.8(N=275) | 90 ± 3.9(N=245) | 92 ± 3.8(N=219) | 89 ± 2.7(N=206) | 86 ± 4.2(N=173) | 100 ± 1.9(N=179) | 85 ± 4.2(N=154) |
|  | **p** | <0.0001 | 1 | <0.0001 | <0.0001 | 1 | <0.0001 | <0.0001 |

Table S. 4: Comparison of differential intracochlear pressure between OSI200 at position 1 versus position 3. Data is shown as mean ± standard deviation. P-values are Bonferroni-corrected for multiple comparisons. P-values that are >1 because of the Bonferroni correction are capped to a value of 1, while p-values lower than 0.0001 are capped to 0.0001 for readability purposes.

|  | **Frequency→**  **Dataset ↓** | **0.5 kHz** | **0.75 kHz** | **1 kHz** | **2 kHz** | **3 kHz** | **4 kHz** | **6 kHz** |
| --- | --- | --- | --- | --- | --- | --- | --- | --- |
| **Head 13** | **OSI200 pos. 1** | 87 ± 3.9(N=274) | 89 ± 4.2(N=231) | 91 ± 3.1(N=228) | 99 ± 1.5(N=210) | 100 ± 0.83(N=193) | 110 ± 0.74(N=180) | 110 ± 0.74(N=168) |
|  | **OSI200 pos. 3** | 87 ± 3.7(N=278) | 88 ± 3.8(N=238) | 85 ± 3.9(N=202) | 84 ± 4(N=195) | 91 ± 2.9(N=193) | 95 ± 2.4(N=177) | 88 ± 3.8(N=162) |
|  | **p** | 0,52 | 0,0041 | <0.0001 | <0.0001 | <0.0001 | <0.0001 | <0.0001 |
| **Head 14** | **OSI200 pos. 1** | 91 ± 3.7(N=209) | 97 ± 2(N=192) | 100 ± 1.8(N=173) | 100 ± 1.4(N=158) | 100 ± 0.87(N=150) | 110 ± 1.3(N=136) | 100 ± 2.3(N=129) |
|  | **OSI200 pos. 3** | 87 ± 4.2(N=103) | 95 ± 2.8(N=122) | 97 ± 2(N=111) | 100 ± 1.4(N=106) | 100 ± 0.84(N=98) | 110 ± 0.63(N=92) | 91 ± 4.2(N=82) |
|  | **p** | <0.0001 | <0.0001 | <0.0001 | <0.0001 | <0.0001 | <0.0001 | <0.0001 |
| **Head 15** | **OSI200 pos. 1** | 84 ± 4.2(N=280) | 86 ± 3.8(N=230) | 90 ± 3.8(N=226) | 86 ± 3.6(N=202) | 94 ± 2.2(N=191) | 100 ± 1.3(N=179) | 100 ± 0.79(N=166) |
|  | **OSI200 pos. 3** | 81 ± 4.2(N=232) | 81 ± 3.6(N=203) | 89 ± 4.2(N=221) | 86 ± 4(N=187) | 83 ± 3.8(N=182) | 100 ± 1.9(N=179) | 85 ± 3.5(N=159) |
|  | **p** | <0.0001 | <0.0001 | 0,099 | 1 | <0.0001 | <0.0001 | <0.0001 |
| **Head 16** | **OSI200 pos. 1** | 85 ± 4.4(N=268) | 90 ± 4(N=237) | 85 ± 4(N=214) | 86 ± 3.6(N=200) | 87 ± 3.8(N=182) | 96 ± 2(N=177) | 88 ± 3.2(N=165) |
|  | **OSI200 pos. 3** | 82 ± 4.3(N=249) | 87 ± 4(N=241) | 91 ± 3.9(N=224) | 84 ± 3.9(N=191) | 82 ± 3.8(N=176) | 100 ± 4.9(N=178) | 96 ± 2.6(N=165) |
|  | **p** | <0.0001 | <0.0001 | <0.0001 | <0.0001 | <0.0001 | <0.0001 | <0.0001 |

### Device type

Table S. 5: Comparison of promontory velocity between OSI200 at position 1 versus Baha position 3. Data is shown as mean ± standard deviation. P-values are Bonferroni-corrected for multiple comparisons. P-values that are >1 because of the Bonferroni correction are capped to a value of 1, while p-values lower than 0.0001 are capped to 0.0001 for readability purposes.

|  | **Frequency→**  **Dataset ↓** | **0.5 kHz** | **0.75 kHz** | **1 kHz** | **2 kHz** | **3 kHz** | **4 kHz** | **6 kHz** |
| --- | --- | --- | --- | --- | --- | --- | --- | --- |
| **Head 13** | **OSI200 pos. 1** | 0.066 ± 0.00023 (N=288) | 0.047 ± 0.00011 (N=255) | 0.041 ± 0.00033 (N=228) | 0.046 ± 0.00061 (N=210) | 0.024 ± 3.6e-05 (N=193) | 0.033 ± 3.4e-05 (N=180) | 0.031 ± 6.4e-05 (N=168) |
|  | **Baha pos. 3** | 0.048 ± 0.00015 (N=290) | 0.057 ± 9.5e-05 (N=256) | 0.021 ± 3.7e-05 (N=230) | 0.021 ± 4.3e-05 (N=209) | 0.011 ± 1.2e-05 (N=195) | 0.015 ± 5e-05 (N=180) | 0.011 ± 4.3e-05 (N=168) |
|  | **p** | <0.0001 | <0.0001 | <0.0001 | <0.0001 | <0.0001 | <0.0001 | <0.0001 |
| **Head 14** | **OSI200 pos. 1** | 0.054 ± 0.0001 (N=209) | 0.058 ± 0.00013 (N=192) | 0.067 ± 0.00015 (N=173) | 0.053 ± 3.2e-05 (N=158) | 0.027 ± 9.7e-06 (N=150) | 0.022 ± 9e-06 (N=136) | 0.025 ± 5.7e-06 (N=129) |
|  | **Baha pos. 3** | 0.038 ± 2.5e-05 (N=129) | 0.055 ± 4.3e-05 (N=120) | 0.031 ± 1.9e-05 (N=104) | 0.02 ± 1.1e-05 (N=103) | 0.013 ± 7.5e-06 (N=90) | 0.012 ± 1.4e-05 (N=90) | 0.0091 ± 6.2e-06 (N=85) |
|  | **p** | <0.0001 | <0.0001 | <0.0001 | <0.0001 | <0.0001 | <0.0001 | <0.0001 |
| **Head 15** | **OSI200 pos. 1** | 0.05 ± 0.00024 (N=289) | 0.036 ± 1.8e-05 (N=255) | 0.057 ± 1.5e-05 (N=229) | 0.02 ± 1.5e-05 (N=208) | 0.016 ± 1.2e-05 (N=191) | 0.013 ± 1.3e-05 (N=179) | 0.024 ± 1.9e-05 (N=166) |
|  | **Baha pos. 3** | 0.034 ± 0.013 (N=293) | 0.013 ± 0.0041 (N=256) | 0.015 ± 0.00086 (N=227) | 0.0067 ± 0.0016 (N=208) | 0.0057 ± 0.002 (N=191) | 0.0091 ± 0.00084 (N=178) | 0.0073 ± 0.00086 (N=166) |
|  | **p** | <0.0001 | <0.0001 | <0.0001 | <0.0001 | <0.0001 | <0.0001 | <0.0001 |
| **Head 16** | **OSI200 pos. 1** | 0.034 ± 0.00063 (N=287) | 0.02 ± 0.0012 (N=252) | 0.042 ± 0.00011 (N=227) | 0.024 ± 0.00034 (N=207) | 0.016 ± 0.00016 (N=190) | 0.024 ± 0.00066 (N=177) | 0.027 ± 0.00021 (N=166) |
|  | **Baha pos. 3** | 0.04 ± 0.00019 (N=289) | 0.026 ± 0.00016 (N=255) | 0.0095 ± 3.5e-05 (N=225) | 0.0079 ± 9.8e-05 (N=208) | 0.0032 ± 1.7e-05 (N=190) | 0.0023 ± 1.3e-05 (N=177) | 0.0098 ± 0.00012 (N=166) |
|  | **p** | <0.0001 | <0.0001 | <0.0001 | <0.0001 | <0.0001 | <0.0001 | <0.0001 |

Table S. 6: Comparison of differential intracochlear pressure between OSI200 at position 1 versus Baha position 3. Data is shown as mean ± standard deviation. P-values are Bonferroni-corrected for multiple comparisons. P-values that are >1 because of the Bonferroni correction are capped to a value of 1, while p-values lower than 0.0001 are capped to 0.0001 for readability purposes.

|  | **Frequency→**  **Dataset ↓** | **0.5 kHz** | **0.75 kHz** | **1 kHz** | **2 kHz** | **3 kHz** | **4 kHz** | **6 kHz** |
| --- | --- | --- | --- | --- | --- | --- | --- | --- |
| **Head 13** | **OSI200 pos. 1** | 87 ± 3.9(N=274) | 89 ± 4.2(N=231) | 91 ± 3.1(N=228) | 99 ± 1.5(N=210) | 100 ± 0.83(N=193) | 110 ± 0.74(N=180) | 110 ± 0.74(N=168) |
|  | **Baha pos. 3** | 82 ± 4.1(N=261) | 91 ± 3.1(N=256) | 84 ± 3.6(N=210) | 80 ± 3.5(N=179) | 95 ± 2.1(N=195) | 100 ± 1.6(N=180) | 99 ± 1.2(N=168) |
|  | **p** | <0.0001 | <0.0001 | <0.0001 | <0.0001 | <0.0001 | <0.0001 | <0.0001 |
| **Head 14** | **OSI200 pos. 1** | 91 ± 3.7(N=209) | 97 ± 2(N=192) | 100 ± 1.8(N=173) | 100 ± 1.4(N=158) | 100 ± 0.87(N=150) | 110 ± 1.3(N=136) | 100 ± 2.3(N=129) |
|  | **Baha pos. 3** | 91 ± 3.3(N=127) | 98 ± 3.2(N=120) | 98 ± 2.2(N=104) | 97 ± 2(N=103) | 94 ± 3.6(N=90) | 100 ± 3.4(N=88) | 93 ± 2.8(N=85) |
|  | **p** | 1 | 0,0011 | 0,000001 | <0.0001 | <0.0001 | <0.0001 | <0.0001 |
| **Head 15** | **OSI200 pos. 1** | 84 ± 4.2(N=280) | 86 ± 3.8(N=230) | 90 ± 3.8(N=226) | 86 ± 3.6(N=202) | 94 ± 2.2(N=191) | 100 ± 1.3(N=179) | 100 ± 0.79(N=166) |
|  | **Baha pos. 3** | 82 ± 4.1(N=265) | 85 ± 3.6(N=237) | 82 ± 3.3(N=202) | 82 ± 3.4(N=176) | 81 ± 3.7(N=162) | 89 ± 3.5(N=173) | 83 ± 3.4(N=163) |
|  | **p** | <0.0001 | 0,0039 | <0.0001 | <0.0001 | <0.0001 | <0.0001 | <0.0001 |
| **Head 16** | **OSI200 pos. 1** | 85 ± 4.4(N=268) | 90 ± 4(N=237) | 85 ± 4(N=214) | 86 ± 3.6(N=200) | 87 ± 3.8(N=182) | 96 ± 2(N=177) | 88 ± 3.2(N=165) |
|  | **Baha pos. 3** | 86 ± 3.8(N=274) | 98 ± 2.4(N=255) | 92 ± 3.1(N=223) | 87 ± 4.3(N=200) | 93 ± 3.2(N=190) | 98 ± 3.9(N=175) | 97 ± 4.4(N=127) |
|  | **p** | 1 | <0.0001 | <0.0001 | 0,0013 | <0.0001 | <0.0001 | <0.0001 |

### Cortical bone undergrowth – Promontory velocity

Table S. 7: Comparison of promontory velocity between OSI200 at position 1 with a non-cemented versus a half-cemented actuator. Data is shown as mean ± standard deviation. P-values are Bonferroni-corrected for multiple comparisons. P-values that are >1 because of the Bonferroni correction are capped to a value of 1, while p-values lower than 0.0001 are capped to 0.0001 for readability purposes.

|  | **Frequency→**  **Dataset ↓** | **0.5 kHz** | **0.75 kHz** | **1 kHz** | **2 kHz** | **3 kHz** | **4 kHz** | **6 kHz** |
| --- | --- | --- | --- | --- | --- | --- | --- | --- |
| **Head 13** | **Not cemented** | 0.066 ± 0.00023 (N=288) | 0.047 ± 0.00011 (N=255) | 0.041 ± 0.00033 (N=228) | 0.046 ± 0.00061 (N=210) | 0.024 ± 3.6e-05 (N=193) | 0.033 ± 3.4e-05 (N=180) | 0.031 ± 6.4e-05 (N=168) |
|  | **Partially cemented** | 0.07 ± 6.5e-05 (N=293) | 0.046 ± 4.5e-05 (N=259) | 0.041 ± 1.6e-05 (N=228) | 0.046 ± 1.3e-05 (N=208) | 0.025 ± 1.5e-05 (N=193) | 0.038 ± 3.3e-05 (N=177) | 0.032 ± 4.9e-05 (N=166) |
|  | **p** | <0.0001 | <0.0001 | <0.0001 | <0.0001 | <0.0001 | <0.0001 | <0.0001 |
| **Head 14** | **Not cemented** | 0.054 ± 0.0001 (N=209) | 0.058 ± 0.00013 (N=192) | 0.067 ± 0.00015 (N=173) | 0.053 ± 3.2e-05 (N=158) | 0.027 ± 9.7e-06 (N=150) | 0.022 ± 9e-06 (N=136) | 0.025 ± 5.7e-06 (N=129) |
|  | **Partially cemented** | 0.054 ± 0.00012 (N=130) | 0.061 ± 2e-05 (N=112) | 0.066 ± 2.3e-05 (N=104) | 0.052 ± 4.6e-05 (N=97) | 0.026 ± 1.4e-05 (N=96) | 0.026 ± 2.6e-05 (N=87) | 0.017 ± 1.3e-05 (N=82) |
|  | **p** | <0.0001 | <0.0001 | <0.0001 | <0.0001 | <0.0001 | <0.0001 | <0.0001 |
| **Head 15** | **Not cemented** | 0.05 ± 0.00024 (N=289) | 0.036 ± 1.8e-05 (N=255) | 0.057 ± 1.5e-05 (N=229) | 0.02 ± 1.5e-05 (N=208) | 0.016 ± 1.2e-05 (N=191) | 0.013 ± 1.3e-05 (N=179) | 0.024 ± 1.9e-05 (N=166) |
|  | **Partially cemented** | 0.049 ± 0.00083 (N=288) | 0.038 ± 6.8e-05 (N=255) | 0.058 ± 5.6e-05 (N=228) | 0.021 ± 5.9e-05 (N=208) | 0.016 ± 0.0001 (N=190) | 0.015 ± 0.00014 (N=177) | 0.025 ± 4.2e-05 (N=168) |
|  | **p** | <0.0001 | <0.0001 | <0.0001 | <0.0001 | <0.0001 | <0.0001 | <0.0001 |
| **Head 16** | **Not cemented** | 0.034 ± 0.0035 (N=283) | 0.023 ± 0.003 (N=253) | 0.043 ± 0.0016 (N=227) | 0.026 ± 0.0029 (N=204) | 0.016 ± 0.001 (N=189) | 0.024 ± 0.0012 (N=177) | 0.027 ± 0.0013 (N=165) |
|  | **Partially cemented** | 0.036 ± 0.0027 (N=287) | 0.023 ± 0.0039 (N=253) | 0.039 ± 0.0047 (N=227) | 0.027 ± 0.0012 (N=207) | 0.016 ± 0.0013 (N=189) | 0.021 ± 0.00078 (N=177) | 0.015 ± 0.0019 (N=164) |
|  | **p** | <0.0001 | 1 | <0.0001 | 0,0084 | 1 | <0.0001 | <0.0001 |

Table S. 8: Comparison of promontory velocity between OSI200 position 1 with a non-cemented versus a completely cemented actuator. Data is shown as mean ± standard deviation. P-values are Bonferroni-corrected for multiple comparisons. P-values that are >1 because of the Bonferroni correction are capped to a value of 1, while p-values lower than 0.0001 are capped to 0.0001 for readability purposes.

|  | **Frequency→**  **Dataset ↓** | **0.5 kHz** | **0.75 kHz** | **1 kHz** | **2 kHz** | **3 kHz** | **4 kHz** | **6 kHz** |
| --- | --- | --- | --- | --- | --- | --- | --- | --- |
| **Head 13** | **Not cemented** | 0.066 ± 0.00023 (N=288) | 0.047 ± 0.00011 (N=255) | 0.041 ± 0.00033 (N=228) | 0.046 ± 0.00061 (N=210) | 0.024 ± 3.6e-05 (N=193) | 0.033 ± 3.4e-05 (N=180) | 0.031 ± 6.4e-05 (N=168) |
|  | **Completely cemented** | 0.065 ± 0.00012 (N=294) | 0.043 ± 7.6e-05 (N=259) | 0.037 ± 5.4e-05 (N=228) | 0.041 ± 8.3e-05 (N=211) | 0.021 ± 2.2e-05 (N=194) | 0.03 ± 8.4e-05 (N=178) | 0.026 ± 6.3e-05 (N=167) |
|  | **p** | <0.0001 | <0.0001 | <0.0001 | <0.0001 | <0.0001 | <0.0001 | <0.0001 |
| **Head 14** | **Not cemented** | 0.054 ± 0.0001 (N=209) | 0.058 ± 0.00013 (N=192) | 0.067 ± 0.00015 (N=173) | 0.053 ± 3.2e-05 (N=158) | 0.027 ± 9.7e-06 (N=150) | 0.022 ± 9e-06 (N=136) | 0.025 ± 5.7e-06 (N=129) |
|  | **Completely cemented** | 0.055 ± 4e-05 (N=130) | 0.062 ± 1.7e-05 (N=120) | 0.064 ± 1.8e-05 (N=110) | 0.052 ± 8.9e-06 (N=103) | 0.026 ± 1e-05 (N=96) | 0.025 ± 1.1e-05 (N=92) | 0.023 ± 8.5e-06 (N=86) |
|  | **p** | <0.0001 | <0.0001 | <0.0001 | <0.0001 | <0.0001 | <0.0001 | <0.0001 |
| **Head 15** | **Not cemented** | 0.05 ± 0.00024 (N=289) | 0.036 ± 1.8e-05 (N=255) | 0.057 ± 1.5e-05 (N=229) | 0.02 ± 1.5e-05 (N=208) | 0.016 ± 1.2e-05 (N=191) | 0.013 ± 1.3e-05 (N=179) | 0.024 ± 1.9e-05 (N=166) |
|  | **Completely cemented** | 0.047 ± 0.0015 (N=290) | 0.035 ± 0.0017 (N=250) | 0.057 ± 0.0028 (N=228) | 0.021 ± 0.0016 (N=207) | 0.016 ± 0.0032 (N=190) | 0.014 ± 0.0046 (N=178) | 0.022 ± 0.0045 (N=164) |
|  | **p** | <0.0001 | 0,00011 | 0,13 | <0.0001 | 0,74 | 0,026 | 0,0032 |
| **Head 16** | **Not cemented** | 0.034 ± 0.0035 (N=283) | 0.023 ± 0.003 (N=253) | 0.043 ± 0.0016 (N=227) | 0.026 ± 0.0029 (N=204) | 0.016 ± 0.001 (N=189) | 0.024 ± 0.0012 (N=177) | 0.027 ± 0.0013 (N=165) |
|  | **Completely cemented** | 0.037 ± 0.00061 (N=285) | 0.023 ± 0.00029 (N=252) | 0.044 ± 4.5e-05 (N=227) | 0.027 ± 4.5e-05 (N=205) | 0.016 ± 2.3e-17 (N=191) | 0.028 ± 0.00011 (N=176) | 0.016 ± 3.1e-05 (N=163) |
|  | **p** | <0.0001 | 0,24 | <0.0001 | 0,11 | <0.0001 | <0.0001 | <0.0001 |

### Cortical bone undergrowth – Differential pressure

Table S. 9: Comparison of differential intracochlear pressure between OSI200 at position 1 with a non-cemented versus a half-cemented actuator. Data is shown as mean ± standard deviation. P-values are Bonferroni-corrected for multiple comparisons. P-values that are >1 because of the Bonferroni correction are capped to a value of 1, while p-values lower than 0.0001 are capped to 0.0001 for readability purposes.

|  | **Frequency→**  **Dataset ↓** | **0.5 kHz** | **0.75 kHz** | **1 kHz** | **2 kHz** | **3 kHz** | **4 kHz** | **6 kHz** |
| --- | --- | --- | --- | --- | --- | --- | --- | --- |
| **Head 13** | **Not cemented** | 87 ± 3.9(N=274) | 89 ± 4.2(N=231) | 91 ± 3.1(N=228) | 99 ± 1.5(N=210) | 100 ± 0.83(N=193) | 110 ± 0.74(N=180) | 110 ± 0.74(N=168) |
|  | **Partially cemented** | 87 ± 4.1(N=283) | 90 ± 4.2(N=240) | 91 ± 3.2(N=227) | 96 ± 2(N=208) | 97 ± 2(N=193) | 100 ± 2.3(N=177) | 99 ± 1.6(N=166) |
|  | **p** | 1 | 0,2 | 1 | <0.0001 | <0.0001 | <0.0001 | <0.0001 |
| **Head 14** | **Not cemented** | 91 ± 3.7(N=209) | 97 ± 2(N=192) | 100 ± 1.8(N=173) | 100 ± 1.4(N=158) | 100 ± 0.87(N=150) | 110 ± 1.3(N=136) | 100 ± 2.3(N=129) |
|  | **Partially cemented** | 83 ± 3.9(N=119) | 83 ± 3.7(N=91) | 87 ± 2.8(N=102) | 92 ± 1.3(N=97) | 90 ± 2.3(N=96) | 100 ± 1.3(N=87) | 95 ± 1.6(N=82) |
|  | **p** | <0.0001 | <0.0001 | <0.0001 | <0.0001 | <0.0001 | <0.0001 | <0.0001 |
| **Head 15** | **Not cemented** | 84 ± 4.2(N=280) | 86 ± 3.8(N=230) | 90 ± 3.8(N=226) | 86 ± 3.6(N=202) | 94 ± 2.2(N=191) | 100 ± 1.3(N=179) | 100 ± 0.79(N=166) |
|  | **Partially cemented** | 83 ± 4.2(N=259) | 85 ± 3.8(N=234) | 88 ± 3.6(N=212) | 90 ± 2.9(N=208) | 92 ± 3.6(N=185) | 99 ± 2.5(N=177) | 89 ± 3.4(N=167) |
|  | **p** | <0.0001 | 0,24 | <0.0001 | <0.0001 | <0.0001 | <0.0001 | <0.0001 |
| **Head 16** | **Not cemented** | 87 ± 5.7(N=150) | 85 ± 4(N=225) | 99 ± 1.4(N=227) | 86 ± 4.4(N=185) | 91 ± 3.1(N=188) | 98 ± 2.4(N=177) | 87 ± 3.2(N=161) |
|  | **Partially cemented** | 88 ± 4.1(N=260) | 86 ± 3.9(N=229) | 85 ± 3.7(N=195) | 89 ± 3.3(N=206) | 99 ± 2.1(N=189) | 110 ± 3.1(N=177) | 88 ± 3.9(N=164) |
|  | **p** | 1 | <0.0001 | <0.0001 | <0.0001 | <0.0001 | <0.0001 | 0,051 |

Table S. 10: Comparison of differential intracochlear pressure between OSI200 at position 1 with a non-cemented versus a completely cemented actuator. Data is shown as mean ± standard deviation. P-values are Bonferroni-corrected for multiple comparisons. P-values that are >1 because of the Bonferroni correction are capped to a value of 1, while p-values lower than 0.0001 are capped to 0.0001 for readability purposes.

|  | **Frequency→**  **Dataset ↓** | **0.5 kHz** | **0.75 kHz** | **1 kHz** | **2 kHz** | **3 kHz** | **4 kHz** | **6 kHz** |
| --- | --- | --- | --- | --- | --- | --- | --- | --- |
| **Head 13** | **Not cemented** | 87 ± 3.9(N=274) | 89 ± 4.2(N=231) | 91 ± 3.1(N=228) | 99 ± 1.5(N=210) | 100 ± 0.83(N=193) | 110 ± 0.74(N=180) | 110 ± 0.74(N=168) |
|  | **Completely cemented** | 83 ± 4.2(N=279) | 86 ± 3.8(N=241) | 87 ± 4.3(N=212) | 94 ± 2.3(N=211) | 95 ± 2(N=194) | 100 ± 1.7(N=178) | 100 ± 1.4(N=167) |
|  | **p** | <0.0001 | <0.0001 | <0.0001 | <0.0001 | <0.0001 | <0.0001 | <0.0001 |
| **Head 14** | **Not cemented** | 91 ± 3.7(N=209) | 97 ± 2(N=192) | 100 ± 1.8(N=173) | 100 ± 1.4(N=158) | 100 ± 0.87(N=150) | 110 ± 1.3(N=136) | 100 ± 2.3(N=129) |
|  | **Completely cemented** | 84 ± 4.2(N=116) | 90 ± 3(N=120) | 90 ± 2.7(N=108) | 91 ± 3.2(N=103) | 87 ± 3.8(N=91) | 88 ± 4(N=90) | 81 ± 3.7(N=80) |
|  | **p** | <0.0001 | <0.0001 | <0.0001 | <0.0001 | <0.0001 | <0.0001 | <0.0001 |
| **Head 15** | **Not cemented** | 84 ± 4.2(N=280) | 86 ± 3.8(N=230) | 90 ± 3.8(N=226) | 86 ± 3.6(N=202) | 94 ± 2.2(N=191) | 100 ± 1.3(N=179) | 100 ± 0.79(N=166) |
|  | **Completely cemented** | 84 ± 4.7(N=202) | 81 ± 3.6(N=206) | 93 ± 3.5(N=225) | 84 ± 3.9(N=175) | 84 ± 3.9(N=178) | 91 ± 2.8(N=178) | 86 ± 3.1(N=161) |
|  | **p** | 1 | <0.0001 | <0.0001 | <0.0001 | <0.0001 | <0.0001 | <0.0001 |
| **Head 16** | **Not cemented** | 87 ± 5.7(N=150) | 85 ± 4(N=225) | 99 ± 1.4(N=227) | 86 ± 4.4(N=185) | 91 ± 3.1(N=188) | 98 ± 2.4(N=177) | 87 ± 3.2(N=161) |
|  | **Completely cemented** | 81 ± 3.6(N=265) | 92 ± 3.8(N=243) | 96 ± 4.1(N=208) | 90 ± 3.8(N=199) | 95 ± 4.4(N=190) | 100 ± 2(N=176) | 82 ± 3.6(N=148) |
|  | **p** | <0.0001 | <0.0001 | <0.0001 | <0.0001 | <0.0001 | <0.0001 | <0.0001 |

### Importance of the fixation system

Table S. 11: Comparison of promontory velocity between OSI200 position 1 with or without a rigid coupling to the skull. Data is shown as mean ± standard deviation. P-values are Bonferroni-corrected for multiple comparisons. P-values that are >1 because of the Bonferroni correction are capped to a value of 1, while p-values lower than 0.0001 are capped to 0.0001 for readability purposes.

|  | **Frequency→**  **Dataset ↓** | **0.5 kHz** | **0.75 kHz** | **1 kHz** | **2 kHz** | **3 kHz** | **4 kHz** | **6 kHz** |
| --- | --- | --- | --- | --- | --- | --- | --- | --- |
| **Head 10** | **With screw** | 0.038 ± 0.00029 (N=211) | 0.01 ± 0.00025 (N=189) | 0.019 ± 5.2e-17 (N=172) | 0.017 ± 0.00012 (N=159) | 0.0057 ± 0.0028 (N=144) | 0.0071 ± 0.00022 (N=137) | 0.0074 ± 0.00016 (N=129) |
|  | **Without screw** | 0.0085 ± 6.1e-05 (N=288) | 0.01 ± 5.7e-05 (N=257) | 0.015 ± 2.4e-05 (N=227) | 0.0095 ± 8e-06 (N=207) | 0.0028 ± 2.3e-05 (N=191) | 0.0052 ± 6e-05 (N=177) | 0.0024 ± 0.0024 (N=163) |
|  | **p** | <0.0001 | 0,0016 | <0.0001 | <0.0001 | <0.0001 | <0.0001 | <0.0001 |
| **Head 16** | **With screw** | 0.029 ± 4e-05 (N=209) | 0.033 ± 3.8e-05 (N=190) | 0.036 ± 0.00022 (N=168) | 0.034 ± 7.6e-05 (N=157) | 0.015 ± 2e-05 (N=147) | 0.021 ± 5.5e-05 (N=138) | 0.0073 ± 0.0004 (N=130) |
|  | **Without screw** | 0.04 ± 0.00014 (N=197) | 0.0084 ± 2.1e-05 (N=179) | 0.014 ± 2.9e-05 (N=165) | 0.0025 ± 3.6e-06 (N=151) | 0.0011 ± 5.3e-06 (N=143) | 0.0011 ± 1.6e-05 (N=133) | 0.0014 ± 1.5e-05 (N=126) |
|  | **p** | <0.0001 | <0.0001 | <0.0001 | <0.0001 | <0.0001 | <0.0001 | <0.0001 |

Table S. 12: Comparison of promontory velocity between OSI200 position 1 with a rigid or semi-rigid connection to the skull. Data is shown as mean ± standard deviation. P-values are Bonferroni-corrected for multiple comparisons. P-values that are >1 because of the Bonferroni correction are capped to a value of 1, while p-values lower than 0.0001 are capped to 0.0001 for readability purposes.

|  | **Frequency→**  **Dataset ↓** | **0.5 kHz** | **0.75 kHz** | **1 kHz** | **2 kHz** | **3 kHz** | **4 kHz** | **6 kHz** |
| --- | --- | --- | --- | --- | --- | --- | --- | --- |
| **Head 16** | **With screw** | 0.029 ± 4e-05 (N=209) | 0.033 ± 3.8e-05 (N=190) | 0.036 ± 0.00022 (N=168) | 0.034 ± 7.6e-05 (N=157) | 0.015 ± 2e-05 (N=147) | 0.021 ± 5.5e-05 (N=138) | 0.0073 ± 0.0004 (N=130) |
|  | **Modelling clay** | 0.016 ± 3.2e-05 (N=210) | 0.022 ± 8.8e-05 (N=190) | 0.021 ± 2.8e-05 (N=172) | 0.026 ± 3e-05 (N=159) | 0.0065 ± 1.9e-05 (N=146) | 0.0044 ± 2.3e-05 (N=137) | 0.0056 ± 1.9e-05 (N=129) |
|  | **p** | <0.0001 | <0.0001 | <0.0001 | <0.0001 | <0.0001 | <0.0001 | <0.0001 |
